# Highly Stable Silver(I) Complexes with Cyclen-Based Ligands Bearing Sulfide Arms: A Step Toward Silver-111 Labeled Radiopharmaceuticals

**DOI:** 10.1021/acs.inorgchem.0c01405

**Published:** 2020-07-13

**Authors:** Marianna Tosato, Mattia Asti, Marco Dalla Tiezza, Laura Orian, Daniel Häussinger, Raphael Vogel, Ulli Köster, Mikael Jensen, Alberto Andrighetto, Paolo Pastore, Valerio Di Marco

**Affiliations:** † Department of Chemical Sciences, University of Padova, via Marzolo 1, 35131 Padova, Italy; ‡ Radiopharmaceutical Chemistry Section, Nuclear Medicine Unit, AUSL-IRCCS di Reggio Emilia, Viale Risorgimento 80, 42122 Reggio Emilia, Italy; § Department of Chemistry, University of Basel, St. Johannsring 19, 4056, Basel, Switzerland; ∥ Institut Laue-Langevin, 71 avenue des Martyrs CS 20156, 38042 Grenoble Cedex 9, France; ⊥ The Hevesy Laboratory, Department Health Technology, Technical University of Denmark (DTU), Frederiksborgvej 399, 4000, Roskilde, Denmark; # Italian Institute of Nuclear Physics, Legnaro National Laboratories, Viale dell’Università 2, 35020 Legnaro (Padova), Italy

## Abstract

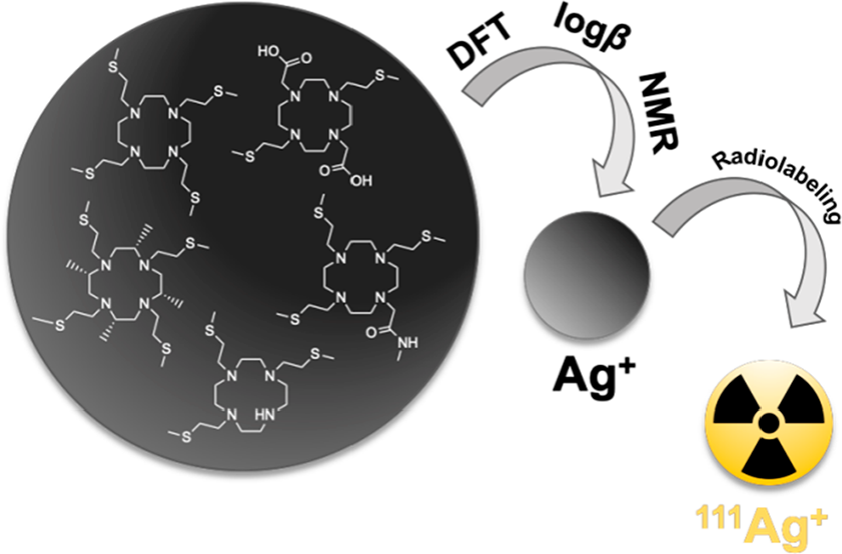

With a half-life of 7.45 days, silver-111 (β_max_ 1.04 MeV, *E*_γ_ 245.4 keV [*I*_γ_ 1.24%], *E*_γ_ 342.1 keV [*I*_γ_ 6.7%]) is a promising candidate for targeted cancer therapy with *β*^*–*^ emitters as well as for associated SPECT imaging. For its clinical use, the development of suitable ligands that form sufficiently stable Ag^+^-complexes *in vivo* is required. In this work, the following sulfur-containing derivatives of tetraazacyclododecane (cyclen) have been considered as potential chelators for silver-111: 1,4,7,10-tetrakis(2-(methylsulfanyl)ethyl)-1,4,7,10-tetraazacyclododecane (DO4S), (2S,5S,8S,11S)-2,5,8,11-tetramethyl-1,4,7,10-tetrakis(2-(methylsulfanyl)ethyl)-1,4,7,10-tetraazacyclododecane (DO4S4Me), 1,4,7-tris(2-(methylsulfanyl)ethyl)-1,4,7,10-tetraazacyclododecane (DO3S), 1,4,7-tris(2-(methylsulfanyl)ethyl)-10-acetamido-1,4,7,10-tetraazacyclododecane (DO3SAm), and 1,7-bis(2-(methylsulfanyl)ethyl)-4,10,diacetic acid-1,4,7,10-tetraazacyclododecane (DO2A2S). Natural Ag^+^ was used in pH/Ag-potentiometric and UV–vis spectrophotometric studies to determine the metal speciation existing in aqueous NaNO_3_ 0.15 M at 25 °C and the equilibrium constants of the complexes, whereas NMR and DFT calculations gave structural insights. Overall results indicated that sulfide pendant arms coordinate Ag^+^ allowing the formation of very stable complexes, both at acidic and physiological pH. Furthermore, radiolabeling, stability in saline phosphate buffer, and metal-competition experiments using the two ligands forming the strongest complexes, DO4S and DO4S4Me, were carried out with [^111^Ag]Ag^+^ and promising results were obtained.

## Abstract 2

Five cyclen derivatives bearing sulfide pendant arms were considered, and their Ag^+^ complex formation was studied in aqueous solution by potentiometry, UV−vis, NMR, radiolabeling experiments with ^111^Ag, and DFT calculations. Overall results indicated that sulfide pendant arms coordinate Ag^+^ allowing the formation of very stable complexes, both at acidic and physiological pH. These ligands might be of interest for the setup of ^111^Ag labeled radiopharmaceuticals.

## Introduction

In the era of precision medicine, targeted radionuclide therapy (TRT) has emerged as a very powerful approach for the treatment of cancer due to its specificity and minimal invasiveness compared to chemotherapy. This strategy relies on a β^–^, α, or Auger radiation-emitting nuclide bound to a biologically active targeting molecule that selectively accumulates into specific disease sites while sparing surrounding healthy cells.^1−4^ In particular, metallic elements provide a large choice of suitable radionuclides, but the stability and hence the biological safety of radiopharmaceuticals containing such kind of radionuclides must be ensured by the formation of stable complexes with a bifunctional chelator (BFC) coupled to the targeting moiety via a covalent linkage.^5−8^ In fact, after injection the radiopharmaceutical must deliver the radionuclide to its cellular, molecular, or biological target without any radiometal loss,^9,10^ which would result in an unwanted dose to the patient and injury to normal tissues.^11^ For such reason, BFCs must display high thermodynamic stability and kinetic inertness toward the radionuclide to prevent dissociation and competition reactions (such as transmetalation by endogenous metals or transchelation by native ligands^9,12^) but also allow mild and rapid labeling conditions, especially if the carrier moiety is a thermally sensitive biomolecule.^13^ Moreover, BFCs not only coordinate the metal radionuclide, but they also modulate the pharmacokinetics of the resulting molecule.

Among the large plethora of *β*^*–*^-emitting metal radionuclides that have been proposed for TRT so far, silver-111 (^111^Ag, *t*_1/2_ 7.47 days) is regarded to be promising due to its medium-energy *β*^*–*^ particles (β_max_ 1.04 MeV) and its two useful low energy γ-rays (*E*_γ_ 245.4 keV [*I*_γ_ 1.24%]; *E*_γ_ 342.1 keV, [*I*_γ_ 6.7%]) suitable for single photon emission computed tomography (SPECT) imaging.^14−18^ An additional positive feature is that the β^+^-emitters silver-103g (*t*_1/2_ 65.7 min, β^+^ 27%, EC 73%) or silver-104g (*t*_1/2_ 69.2 min, β^+^ 15%, EC 85%) could be used as positron emission tomography (PET) imaging analogues.^14,19^ A metal ion that is both diagnostic and therapeutic (i.e., theranostic) allows a more reliable evaluation of the absorbed dose and, consequently, a better indication of the therapeutic activity necessary for the treatment with respect to two different elements, one for diagnosis and one for therapy.^20^ Furthermore, the relatively long half-time of ^111^Ag matches well with the biological half-lives of antibodies (2–3 weeks), making this isotope interesting for use in radioimmunotherapy.^21,22^

The production of ^111^Ag can be accomplished via neutron irradiation of enriched palladium-110 (^110^Pd) targets to give the short-lived palladium-111 (^111^Pd, *t*_1/2_ 23.4 min) by ^110^Pd(n, γ)^111^Pd reaction.^18,23,24^ Short-lived ^111^Pd then decays to ^111^Ag.^16^ Direct production via ^110^Pd(d,n)^111^Pd is also possible with access to medium energy deuteron beams (10–20 MeV).^14^ As an alternative route, the ^111^Ag production via Isotope Separation Online (ISOL) technique is being investigated at the Legnaro National Laboratories of the Italian Institute of Nuclear Physics (ISOLPHARM project).^25,26^

Only limited preclinical applications of ^111^Ag are reported in the literature so far,^15,16^ but to the best of our knowledge no previous research has investigated ^111^Ag for its application in TRT. A key step to attain this goal is to develop suitable ligands that can act as BFCs forming sufficiently stable Ag^+^ complexes under *in vivo* conditions. Silver chelators could be of interest in another important field which is not connected with nuclear medicine. In fact, silver belongs to a group of relatively rare metal ions which represent “bottleneck elements” in the development of green technologies^27,28^ due to their increasing demand and supply shortages.^29^ Effective strategies are needed to ensure the recovery and the recycling of Ag from technological devices which contain this metal in considerable amounts. Available recycling methods have been recently reviewed by Gu et al.^30^ and include acid, thiourea, or thiosulfate leaching, but they suffer from several limitations and are not considered satisfactory. The development of strong Ag^+^ chelators can allow to set up more efficient recycling strategies for this metal.

**Figure 1:**
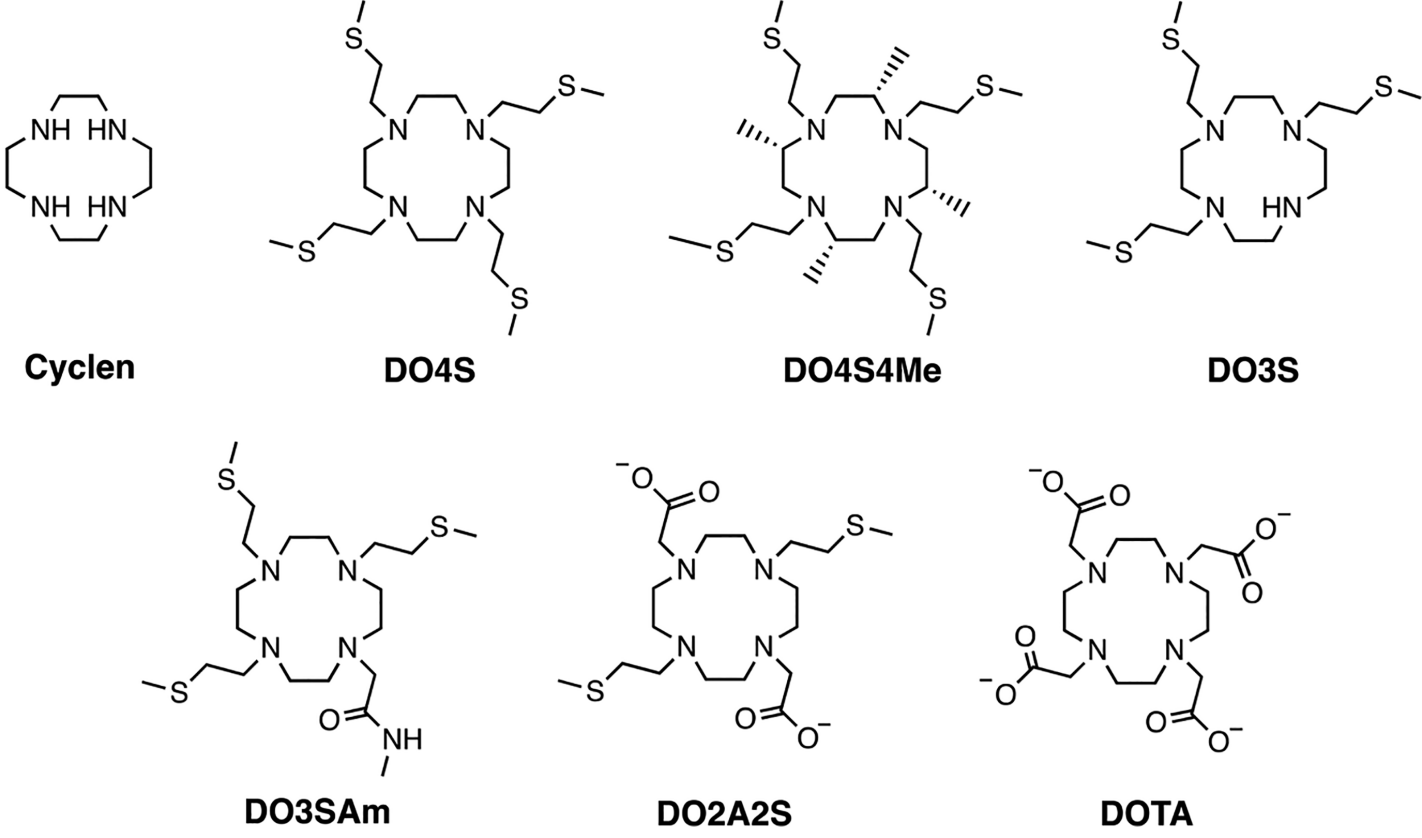
Structure of the chelators discussed in this work. All compounds are shown in their completely deprotonated form.

Tri- and tetraazamacrocyclic ligands with coordinating pendant arms (e.g., DOTA, DO2A, cross-bridged-DO2A, NOTA^31−35^) exhibit both high thermodynamic stability and kinetic inertness toward several metal ions due to their constrained geometries and partially preorganized coordination sites and have been widely investigated in TRT as BFCs for a large variety of radionuclides so far.^2,36^ However, these ligands are not predicted to be the chelators of election for Ag^+^ since its soft character would prevent the formation of stable coordination bonds with hard donor groups like the carboxylates of DOTA and similar analogues. Our research group has recently introduced a series of novel macrocyclic chelators based on the 1,4,7,10-tetraazacyclododecane scaffold (cyclen) with sulfide side arms,^37^ which were able to form very stable complexes with Cd^2+^. A similar affinity is expected with other soft metal ions including Ag^+^. The complexation behavior of 1,4,7,10-tetrakis(2-(methylsulfanyl)ethyl)-1,4,7,10-tetraazacyclododecane (DO4S, Figure 1) with Ag^+^ has been already investigated in aqueous methanol solutions by Mäcke et al., who reported the formation of extremely stable complexes (log *K*_[Ag(DO4S)]_^+^ = 19.64, log *K*_[HAg(DO4S)]_^2+^ = 2.8, 0.1 M KNO_3_, *T* = 25 °C).^38^ However, the mixed organic solvent used therein does not well represent TRT conditions, thus it is worth that the thermodynamic and solution properties of the Ag^+^-DO4S complexes will be studied again in full aqueous solution.

We have identified other sulfur-containing cyclen derivatives, which are shown in Figure 1, to further investigate and gain understanding on the effect of donor atoms and number of S-donors on overall complex stability. 1,4,7-Tris(2-(methylsulfanyl)ethyl)-1,4,7,10-tetraazacyclododecane (DO3S) and 1,7-bis(2-(methylsulfanyl)ethyl)-4,10,diacetic acid-1,4,7,10-tetraazacyclododecane (DO2A2S) bear three and two sulfanyl arms, respectively. In the latter, the two carboxylic groups enhance water solubility and can potentially allow the conjugation to a targeting vector by a covalent link to amino groups. The amido-derivative of DO3S, that is, 1,4,7-tris(2-(methylsulfanyl)ethyl)-10-acetamido-1,4,7,10-tetraazacyclododecane (DO3SAm), was introduced to mimic the behavior of DO3S conjugated to a biological vector. The above listed ligands have been considered also in our previous work.^37^ In this work, a novel additional derivative was considered, (2S,5S,8S,11S)-2,5,8,11-tetramethyl-1,4,7,10-tetrakis(2-(methylsulfanyl)ethyl)-1,4,7,10-tetraazacyclododecane (DO4S4Me). It was designed to enforce the preorganization and enhance the rigidity of the donor atoms by introducing chiral methyl groups on the polyamine backbone. This modification was inspired by the chiral DOTA derivatives recently proposed by Dai et al. which have demonstrated higher stability and faster labeling properties with copper-64 and lutetium-177 compared to the DOTA analogues.^13^

Herein, the complexation behavior of the compounds listed in Figure 1 toward Ag^+^ is presented. DOTA and cyclen were included for comparison purposes, and because their complex formation with Ag^+^ has been hitherto never reported in aqueous solution (data are available only for cyclen and only in some organic solvents^39^). Experimental studies were performed by potentiometry and UV–vis spectrophotometry as well as via nuclear magnetic resonance (1D ^1^H NMR and 2D COSY, NOESY, HMQC). Density functional theory (DFT) calculations provided insight into the structure of selected complexes. In addition to the nonradioactive chemistry, preliminary radiolabeling studies, stability in saline phosphate buffer, Zn^2+^-transmetalation, and metal-competition experiments were performed using [^111^Ag]Ag^+^.

## Experimental Section

### Materials and Methods

All solvent and reagents were purchased from commercial suppliers (Sigma-Aldrich, Fluka, VWR Chemicals) and were used without further purification. 1,4,7,10-Tetraazacyclododecane (cyclen) and 1,4,7,10-tetraazacyclododecane-1,4,7,10-tetraacetic acid (DOTA) were purchased from Chematech. DO4S, DO3S, DO3SAm, DO2A2S, and (2S,5S,8S,11S)-2,5,8,11-tetramethyl-1,4,7,10-tetraazacyclododecane (M4-cyclen) were synthesized according to previously reported procedures.^37,40^

NMR spectra (^1^H 1D, COSY, NOESY, HMQC) were collected at room temperature on Bruker DMX 600, on Bruker 600 Avance III, and on Bruker 400 Avance III HD. Chemical shifts are reported as parts per million (ppm). Spectrophotometric UV–vis measurements were performed on a Cary 60 (Agilent) equipped with a 1 cm path length optical Torlon fiber probe. Analytical and preparative HPLC of DO4S4Me were performed on a Shimadzu LC20 HPLC-system equipped with a prominence UV–vis detector, FRC-10A fraction collector, and a Shimadzu 2020 ESI-MS detector. For analytical and preparative HPLC a ReprosilPur120 ODS-3 3 μm 150 × 3 mm column and a Reprosil-Pur 120 ODS-3 5 μm 30 × 20 mm column were used, respectively. The methods use a binary gradient with solvent A, water + 0.1% trifluoroacetic acid, and solvent B, 90% acetonitrile + 10% water + 0.085% trifluoroacetic acid. Analytical method: flow rate, 1.0 mL/min; oven temperature 40 °C; UV set to 254 and 280 nm; gradient, 2 min at 5% B followed by a gradient over 4 min from 5% B to 100% B. After 8 min, a gradient from 100% B to 5% B over 1 min followed. These conditions were kept constant for another 7 min. Preparative method: flow rate, 10 mL/min; oven temperature 40 °C; UV set to 254 and 280 nm; gradient, 2 min at 5% B followed by a gradient over 7 min from 5% B to 100% B. After 7 min, a gradient from 100% B to 5% B over 1 min followed. These conditions were kept constant for another 2 min. ESI-MS was set at a positive mode and mass spectra were recorded in the 100–1500 *m*/*z* range. A fraction collector was set to the compound mass and a sample volume of 5–10 mL per fraction was collected.

### Synthesis of DO4S4Me

M4-cyclen (50 mg, 0.219 mmol, 1.0 equiv), potassium carbonate (305 mg, 2.19 mmol, 10 equiv), and potassium iodide (10.7 mg, 0.0645 mmol, 0.29 equiv) were suspended in acetonitrile and 2-chloroethyl-methylsulfide (121 mg, 0.110 mL, 1.10 mmol, 5.0 equiv) was added. The suspension was heated to 40 °C for 52 h and was afterward quenched by adding triethylamine (0.5 mL). The suspension was allowed to cool to room temperature and it was filtered. The solvent was evaporated and the crude was purified by preparative HPLC to yield (2S,5S,8S,11S)-2,5,8,11-tetramethyl-1,4,7,10-tetrakis(2-(methylsulfanyl)ethyl)-1,4,7,10-tetraazacyclododecane (DO4S4Me) (103 mg, 0.196 mmol, 90%) as a yellowish oil. ^1^H NMR (600 MHz, acetonitrile-*d*_3_) δ: 3.77–3.60 (m, 8H, H_3a,2_), 3.28–3.05 (m, 8H, H_4_), 2.99–2.83 (m, 8H, H_5_), 2.65–2.59 (m, 4H, H_3b_), 2.18 (s, 12H, H_6_), 1.24 (d, ^3^*J*_HH_ = 6.06 Hz, 12H, H_1_) ppm (Figures S1, S2, and S3). ^13^C NMR (150 MHz, acetonitrile-*d*_3_) δ: 53.86 (C_2_), 52.18 (C_4_), 50.05 (C_3_), 30.43 (C_5_), 15.91 (C_6_), 12.00 (C_1_) ppm. HR-ESI-MS for [M + H]^+^ C_24_H_52_N_4_S_4_*(m*/*z*): calcd. 525.3148; found, 525.3141.

### Thermodynamic Measurements

The potentiometric, UV–vis, and NMR measurements were carried out as reported in detail previously.^37^ Here a brief description is reported.

Automatic potentiometric titrations were performed in aqueous solutions at 25.0 ± 0.1 °C, and the ionic strength was fixed to 0.15 M NaNO_3_ (Carlo Erba, 99% min). The following solutions were prepared and used: 0.1 M HNO_3_ (prepared from the concentrated one (Aristar - VWR Chemicals, 69%) and standardized against Na_2_CO_3_ (Aldrich, 99.95–100.5%)), 0.1 M NaOH (prepared from Fluka, 99% min, and standardized against 0.1 M HNO_3_), ligand ∼3 × 10^–3^ M (with coadded HNO_3_ 1.3 × 10^–2^ M), and Ag^+^ 2 × 10^–2^ M (prepared from AgNO_3_ (Aldrich, >99.98% min)). The ligand concentration in the potentiometric cell varied in the range of 4 × 10^–4^ to 1 × 10^–3^ M, the metal-to-ligand ratios were between 1:1 and 1:2, and the explored pH range was 2–12. Additional potentiometric titrations were performed at a constant pH 2.00 (by HNO_3_) for solutions containing Ag^+^, but the combined glass electrode was replaced by a silver electrode (Crison) and a Ag/AgCl/KCl 3 M double junction reference electrode (Crison).

UV–vis spectrophotometric measurements were performed using the same procedure as that of potentiometric titrations but also an optical fiber was immersed in the titration cell.

The potentiometric and UV–vis data were elaborated with PITMAP program.^41^ The acidity constants are given as p*K*_a_ and refer to the equilibrium H_*h*_L^*n*+^ ⇌ H_*h*–1_L^(*n*–1)+^ + H^+^. The equilibrium constants of complex formation refer to the reactions given in Table 1.

**Table 1:** Stoichiometry and Equilibrium Constants (log β) for the Complexes Formed in Aqueous NaNO_3_ 0.15 M at *T* = 25 °C between Ag^+^ and the Ligands Listed in Figure 1^a^

ligand	equilibrium reaction	logβ
DO4S	Ag^+^ + H^+^ + L ⇌ AgHL^2+^	21.03 ± 0.04^b^
	Ag^+^ + L ⇌ AgL^+^	16.51 ± 0.03
		16.9 ± 0.1^c^
DO4S4Me	Ag^+^ + H^+^ + L ⇌ AgHL^2+^	20.76 ± 0.01^b^
	Ag^+^ + L ⇌ AgL^+^	18.00 ± 0.07
		17.9 ± 0.2^c^
DO3S	Ag^+^ + H^+^ + L ⇌ AgHL^2+^	22.09 ± 0.04^b^
	Ag^+^ + L ⇌ AgL^+^	16.12 ± 0.01
		15.81 ± 0.09^c^
DO3SAm	Ag^+^ + H^+^ + L ⇌ AgHL^2+^	20.16 ± 0.05
	Ag^+^ + L ⇌ AgL^+^	15.48 ± 0.05
DO2A2S	Ag^+^ + 2H^+^ + L^2–^ ⇌ AgH_2_L^+^	22.82 ± 0.09
		23.2 ± 0.5^c^
	Ag^+^ + H^+^ + L^2–^ ⇌ AgHL	19.63 ± 0.06
		19.6 ± 0.3^c^
	Ag^+^ + L^2–^ ⇌ AgL^–^	13.71 ± 0.06
DOTA	Ag^+^ + H^+^ + L^4–^ ⇌ AgHL^2–^	16.6 ± 0.2
	Ag^+^ + L^4–^ ⇌ AgL^3–^	9.1 ± 0.2
Cyclen	Ag^+^ + L ⇌ AgL^+^	6.60 ± 0.02

a L denotes the completely deprotonated ligand form (shown in Figure 1). If not differently stated, values were obtained by pH-potentiometric titrations. The reported uncertainty was obtained by the fitting procedure and represents one standard deviation unit.

b Ag-potentiometric titrations.

c UV–vis spectrophotometric titrations.

^1^H NMR spectra were recorded at room temperature using 3-(trimethylsilyl)propionic acid sodium salt (Sigma-Aldrich, 99%) as internal reference. Spectra were collected in D_2_O (Aldrich, 99.9% D) or H_2_O + 10% D_2_O at about 1 × 10^–3^ M ligand concentration and at a 1:1 metal-to-ligand ratio. Water signal presaturation or excitation sculpting suppression were used, respectively. In pure D_2_O, the “pD” scale was used in the place of the pH scale.^37^

### Density Functional Theory Calculations

All density functional theory (DFT)^42,43^ calculations were performed with the Amsterdam Density Functional (ADF) program.^44−46^ Scalar relativistic effects were accounted for using the zeroth-order regular approximation (ZORA). For geometry optimizations (Table S1) carried out with no symmetry constraint and using analytical gradient techniques, the OPBE^47,48^ density functional was used in combination with the TZP basis set for Ag and DZP basis set for lighter elements.^49^ This potential has proved to provide good structural properties and energies even in the presence of heavy nuclei.^50−52^ All structures were verified by frequency calculations: all normal modes have real frequencies. In order to achieve higher accuracy for energies, single point calculations were performed on the optimized structures using OPBE and the TZ2P basis set for all elements. TZ2P basis set is a large uncontracted set of Slater-type orbitals (STOs). It is of triple-ζ quality and has been augmented with two sets of polarization functions on each atom: 2p and 3d in the case of H, 3d and 4f in the case of C, N, and S, and 5p and 4f in the case of Ag. The frozen-core approximation was employed: up to 1s for C, N, S and up to 3d for Ag. Solvent (water) effects have been accounted using the Conductor-like Screening Model (COSMO).^53−57^ A radius of 1.93 Å and a relative dielectric constant of 78.39 were used. The empirical parameter in the COSMO equation was considered to be 0.0. The radii of the atoms are the classical MM3 radii divided by 1.2.

In order to gain insight into the nature of the bonding between Ag^+^ and the ligands, the activation strain model^58^ was used, which provides a meaningful description of structural and reactivity properties of chemical species.^59−62^ In the activation strain analysis (ASA), the energy relative to the involved fragments (Ag^+^ and ligand), Δ*E*, is decomposed into the strain energy Δ*E*_strain_ and the interaction energy Δ*E*_int_ (eq 1)
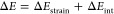
1Δ*E*_strain_ is the energy associated with deforming the fragments from their equilibrium geometry into the geometry they have in the metal complex. It can be divided into a contribution stemming from each fragment. Δ*E*_int_ is the actual interaction energy between the deformed fragments. Δ*E*_int_ can also be further analyzed in the framework of the Kohn–Sham Molecular Orbital (MO) model using a quantitative energy decomposition (EDA) of the bond into electrostatic attraction, Pauli repulsion (or exchange repulsion), and stabilizing orbital interactions (eq 2)
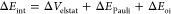
2

### Production and Purification of Silver-111

A 2.54 mg sample of metallic Pd powder, enriched to 98.6% in ^110^Pd (Oak Ridge National Lab, batch 214301) was enclosed in a quartz ampule and irradiated for 4 days in a thermal neutron flux of about 1.1 × 10^15^ cm^–2^ s^–1^ in the beam tube V4 of the high flux reactor at Institut Laue-Langevin in Grenoble, France. Thermal neutron capture on ^110^Pd produces short-lived ^111^Pd which beta-decays with 23.4 min half-life to ^111^Ag. The samples were shipped to Hevesy Lab, Risø, Denmark for radiochemical separation of the no-carrier added ^111^Ag. The purification procedure was performed as reported in literature.^63^

### Radiolabeling Studies with [^111^Ag]Ag^+^

Stock solutions of DO4S and of DO4S4Me were prepared at concentrations of around 1 mg mL^–1^ in deionized water. To evaluate the effect of the ligand concentration, an aliquot of the postprocessed [^111^Ag]Ag^+^ eluate stock solution (∼4 MBq/mL in HCl 1 M, 300 μL) was incubated with an increasing aliquot of each chelator stock solution (varied from 1 to 20 nmol, that is, from 1 × 10^–6^ to 2 × 10^–5^ M) in 1.5 M sodium acetate buffer (pH 4). The influence of the temperature on the reaction yield was evaluated by incubating the reaction mixtures containing [^111^Ag]Ag^+^ 1 MBq, 20 nmol of the ligand at different temperature (RT or 50 °C) in acetate buffer (1.5 M, pH 4) for 5 min. To determine the ligand efficiency at different pH, the reaction mixtures ([^111^Ag]Ag^+^ 1 MBq, 20 nmol of the ligand) were buffered with KH_2_PO_4_ 1.5 M and Na_2_HPO_4_ 1.5 M (pH 7), or diluted with HCl 0.01 M (pH 2).

Radiolabeling was monitored via thin layer chromatography (TLC) using RP-silica gel plates. The TLC were developed by water/methanol (25:75 volume ratio) + ammonium acetate 5% as eluent. Under these conditions, Ag^+^ complexes are retained at the origin (*R*_f_ = 0) whereas free Ag^+^ moves with the solvent front (*R*_f_ = 1). The TLC plate was exposed to a multisensitive medium phosphor screen (PerkinElmer) for 3 min using a Cyclone Plus Storage Phosphor System (PerkinElmer). The stability in PBS and in the presence of Zn^2+^ was checked by diluting the radiolabeled complex solution with an equal volume of PBS and by adding a 5:1 metal-to-ligand (molar ratio) excess of Zn^2+^, respectively. The time-dependent stability was determined after 0, 2, 24, and 48 h in PBS and after 0, 2, and 24 h in the presence of Zn^2+^. Metal competition studies were performed by labeling both ligands with [^111^Ag]Ag^+^ (2 × 10^–5^ M ligand, pH 4, 50 °C, 5 min) in the presence of a 2:1 metal-to-ligand excess of Cu^2+^ or of Cd^2+^.

## Results and Discussion

### Chelators Synthesis

**Scheme 1:**
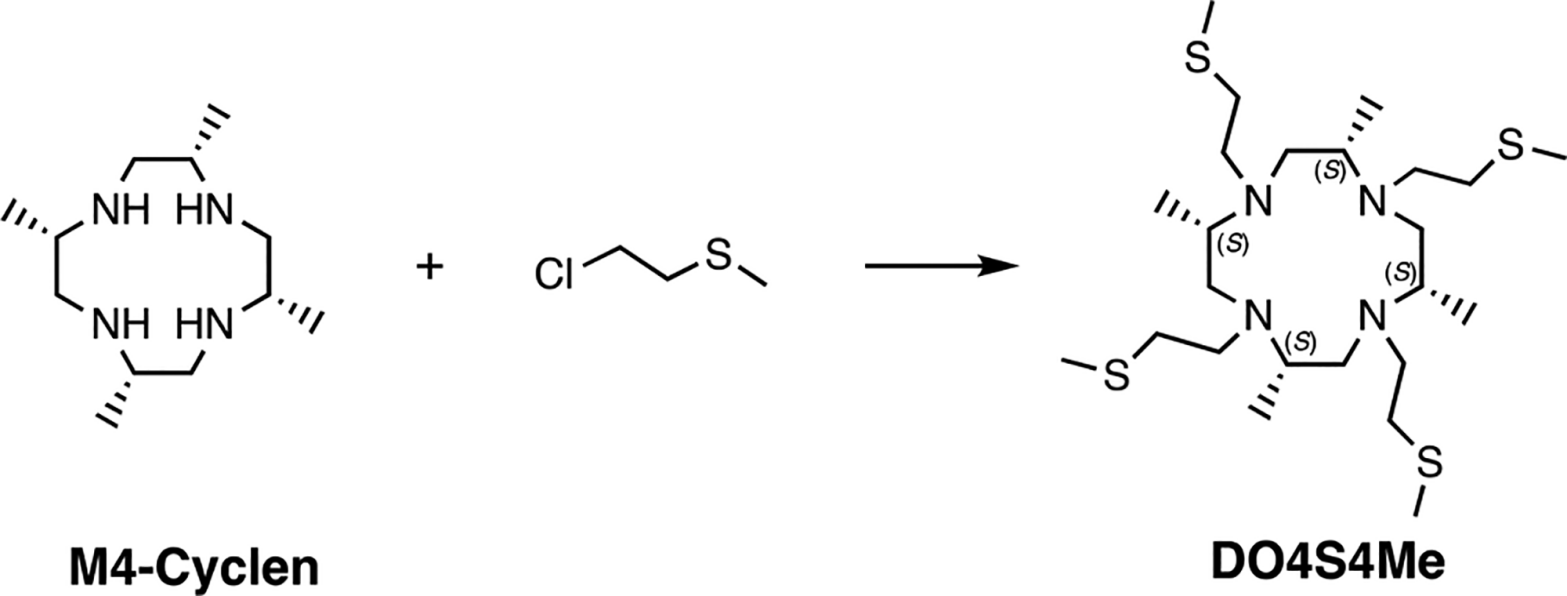
Synthesis Scheme of DO4S4Me Reaction conditions: 2-chloroethyl methyl sulfide (5 equiv), K_2_CO_3_, KI, acetonitrile, 40 °C, 52 h.

DO4S, DO3S, DO3SAm, DO2A2S, and (2S,5S,8S,11S)-2,5,8,11-tetramethyl-1,4,7,10-tetraazacyclododecane (M4-cyclen) were synthesized according to the literature procedures.^37,40^ DO4S4Me was obtained by complete alkylation of M4-cyclen with 2-chloroethyl methyl sulfide in the presence of potassium carbonate in acetonitrile at 40 °C (Scheme 1). The larger steric bulk of M4-cyclen reduced the rate of alkylation when compared to cyclen. Consequently, to speed up the reaction an *in situ* Finkelstein reaction was performed by adding potassium iodide.

### Ag^+^ Solution Thermodynamics

Our group has recently studied the acid–base properties of DO4S (p*K*_H2L_ = 7.29 and p*K*_HL_ = 10.14), DO3S (p*K*_H2L_ = 7.54 and p*K*_HL_ = 10.86), DO3SAm (p*K*_H2L_ = 7.8 and p*K*_HL_ = 10.42), and DO2A2S (p*K*_H3L_ = 3.44 and p*K*_H2L_ + p*K*_HL_ = 18.30) in aqueous NaNO_3_ 0.15 M at *T* = 25 °C.^37^ In the present work, the acidity constants of DO4S4Me were determined in the same conditions by pH-potentiometric titration, finding p*K*_H2L_ = 7.87 ± 0.20 and p*K*_HL_ = 10.458 ± 0.070, respectively. Roughly similar values were obtained by spectrophotometric titrations (Figure S4). Like for the other sulfanyl derivatives,^37^ the H_4_L^4+^ and H_3_L^3+^ forms of DO4S4Me deprotonate at pH values below 2, justifying the only two proton additions detected from our measurements. The minor acidity of DO4S4Me with respect to its achiral analogue DO4S (e.g., p*K*_H2L_ = 7.29 for the latter, p*K*_H2L_ = 7.87 for the former) may arise from the structural rigidity induced by the methyl substituents (see below), which can stabilize not only the metal complexation but also the proton binding.

The equilibrium constants of the Ag^+^-ligand complexes were determined by potentiometry and in some cases by UV–vis spectroscopy as well. The constants for DO3SAm, DO2A2S, DOTA, and cyclen were all accessible by normal pH-potentiometric titrations, whereas additional potentiometric measurements with a silver electrode were required to obtain reliable equilibrium constants for DO4S, DO4S4Me, and DO3S. This was due to the very high complex stability which caused the complexation to start at very low pH (<2). An analogous result was observed for the Cd^2+^ complexes^37^ and it was attributed to the absence of competitive protonation equilibria on SCH_3_, allowing this functional group to strongly bind to metal ions also at very acidic pH. The determined equilibrium constants are summarized in Table 1.

**Figure 2:**
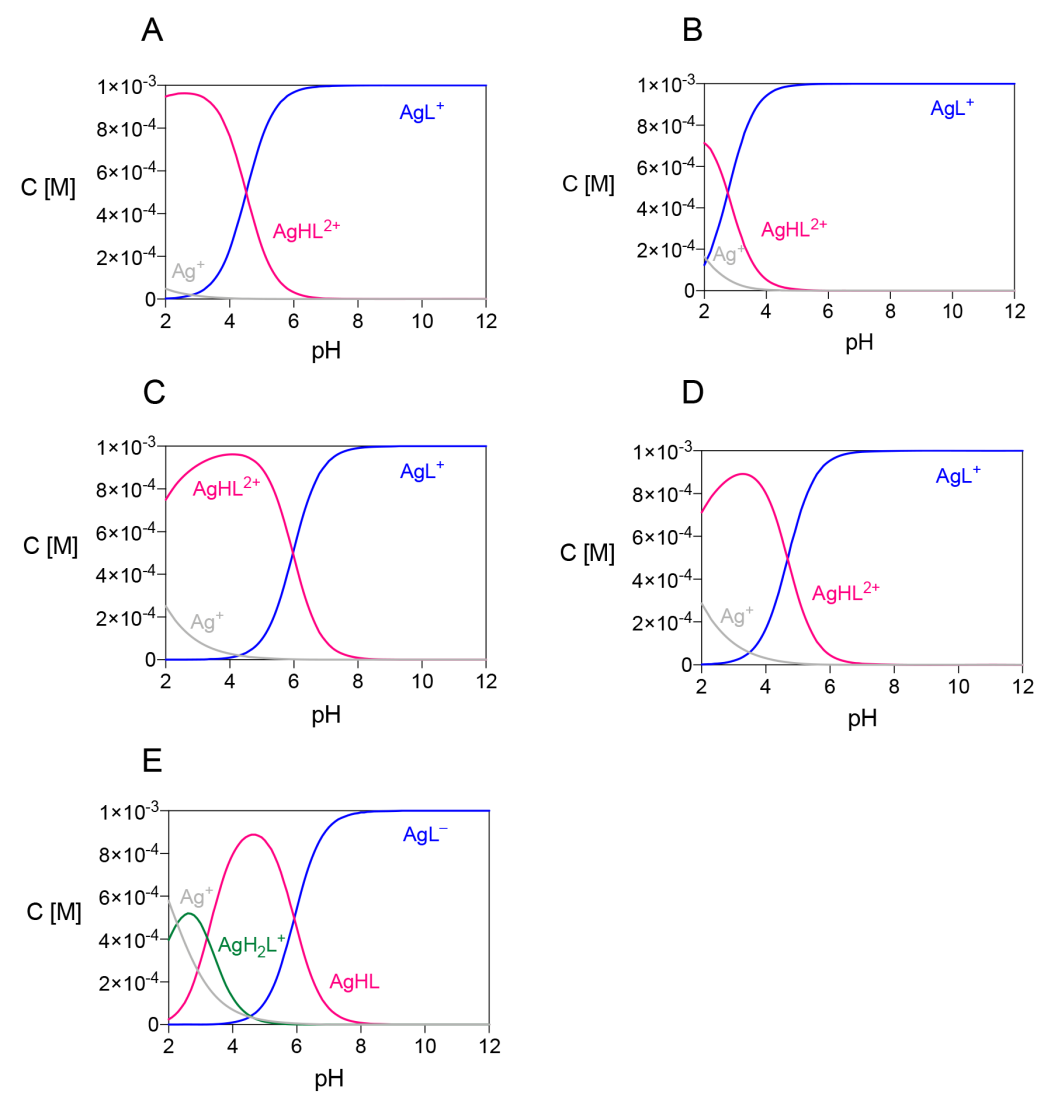
Distribution diagrams of the Ag^+^ complexes formed by (A) DO4S, (B) DO4S4Me, (C) DO3S, (D) DO3SAm, and (E) DO2A2S. (*C*_Ag_ = *C*_ligand_ = 1 × 10^–3^ M).

The distribution diagrams in Figure 2 confirm the complex formation at very acidic pH. By increasing the pH, the successive formation of AgHL^2+^ and AgL^+^ for DO4S, DO4S4Me, DO3S, DO3SAm, and of AgH_2_L^+^, AgHL and AgL^–^ for DO2A2S, takes place. Diagrams change with overall ligand and metal concentration, but at physiological pH the main complex is always the fully deprotonated one (AgL). In the case of Cd^2+^, no protonated complexes were detected (except for the CdHL^+^ complex formed by DO2A2S);^37^ this different behavior can be attributed to the larger charge of Cd^2+^ with respect to Ag^+^, so that deprotonation is promoted with the former metal ion.

**Figure 3:**
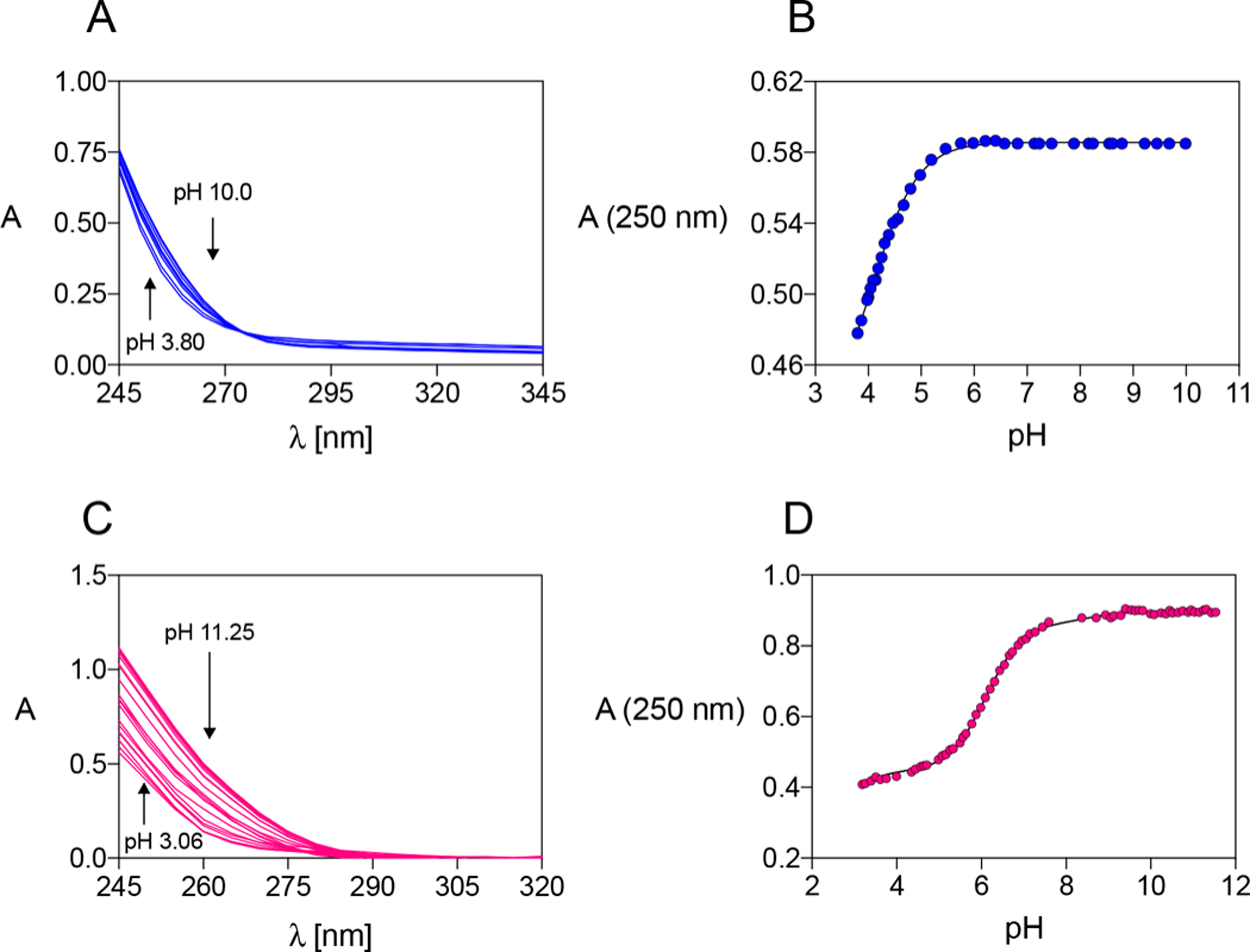
(A) UV–visible spectra of solutions containing Ag^+^ and DO4S (*C*_Ag_ = *C*_DO4S_ = 2.7 × 10^–4^ M, 3.80 ≤ pH ≤ 10.00); (B) corresponding experimental points and fitting line of absorbance vs pH at λ = 250 nm; (C) UV–visible spectra of solutions containing Ag^+^ and DO2A2S (*C*_Ag_ = *C*_DO2A2S_ = 3.7 × 10^–4^ M, 3.06 ≤ pH ≤ 11.25); (D) corresponding experimental points and fitting line of absorbance vs pH at λ = 250 nm.

The equilibrium constants for Ag^+^-DO4S, Ag^+^-DO4S4Me, and Ag^+^-DO2A2S complexes were also confirmed by UV–vis spectrophotometric titrations (Table 1). Representative UV–vis spectra of solutions containing ligand and AgNO_3_ at various pH values are reported in Figures 3A,C and S5. For all ligands, the presence of Ag^+^ causes a bathochromic and hyperchromic effect on the absorption band, which is accountable for the complexation event and was attributed to a charge-transfer transition. The UV–vis band changed also according to the proton content of the complexes, exhibiting a redshift and a general absorbance increase when the pH becomes more basic. The absorbance versus pH graphs at a selected wavelength are shown in Figures 3B,D and S5. It is noteworthy that these graphs well resemble the distribution diagrams of the deprotonated complex AgL of all ligands (for example, compare the blue curve in Figure 2A with experimental points in Figure 3B). This indicates that the main chromophore at the considered wavelength corresponds to AgL (e.g., for Ag^+^-DO4S ε_(250 nm, pH 4.00)_ ≈ 1.8 × 10^3^ L mol^–1^ cm^–1^ and ε_(250 nm, pH 10.00)_ ≈ 2.2 × 10^3^ L mol^–1^ cm^–1^; for Ag^+^-DO2A2S ε_(250 nm, pH 10.08)_ ≈ 2.4 × 10^3^ L mol^–1^ cm^–1^ and ε_(250 nm, pH 4.35)_ ≈ 1.2 × 10^3^ L mol^–1^ cm^–1^).

As already reported, one of the features that a ligand has to possess to represent a good BFC for TRT is the formation of very stable complexes with the radionuclide of interest. In order to compare the Ag^+^ complex stability of the examined ligands, our thermodynamic data were used to compute the pAg values, that is, the cologarithm of free metal concentration (−log[Ag^+^]): the higher the pAg is , the stronger the complex is.^64−66^ The pAg values determined at selected pH values are summarized in Table 2. According to these values, DO4S4Me forms the most stable complexes with Ag^+^, especially at physiological pH. The pAg for this chelator is 0.8 log unit higher than that for DO4S, suggesting that the added chiral modification on the cyclen ring allows a preorganization of the geometry in a more favorable conformation of the donor atoms for metal ion binding, thereby increasing the stability of the resulting metal complex. A relatively small pAg difference of about one log unit can be observed also between DO4S and DO3S that can be assigned to a statistical effect taking place in the presence of the fourth thioether chain which promotes the complexation in DO4S. The introduction of the amide pendant arm into the DO3S frame does not remarkably reduce the thermodynamic stability of the complex (the pAg of DO3SAm is 0.4 log units lower than that of DO3S), suggesting that the pendant arm, linking the BFC to the targeting moiety, only slightly affects the complex formation. The molecule with only two sulfanyl arms (DO2A2S) forms Ag^+^ complexes which are around 2 log units less stable than those bearing three arms (DO3S and DO3SAm), but its pAg value at physiological pH is still 4–5 log units larger than those of DOTA and cyclen. The trend shown in Table 2 strongly suggests that complex stability is governed by the number of sulfide donating groups.

**Table 2:** pAg Values Calculated for *C*_Ag_ = 1 × 10^–6^ M, *C*_L_ = 1 × 10^–5^ M^67^ at 25°C, and at Various pH Values

	pAg		
ligand	pH 4.0	pH 6.0	pH 7.4
DO4S	8.7	12.0	14.5
DO4S4Me	8.6	12.6	15.3
DO3S	8.7	11.0	13.3
DO3SAm	7.0	10.2	12.9
DO2A2S	6.4	8.6	11.2
DOTA	6.0	6.1	6.9
cyclen	6.0	6.0	6.0

### DFT Structural Analysis

DFT calculations have been carried out for the AgL^+^ complexes formed by DO4S, DO4S4Me, and DO3S, and for the AgHL^2+^ complex formed by DO4S. DO4S and DO3S were considered to evaluate the coordination role of the sulfanyl side chains, whereas for DO4S4Me a different stiffness of the cyclen backbone is expected due to the presence of methyl groups. The crystallographic structure deposited with the NAXJIF identifier in the Cambridge Structural Database (CSD)^68^ was used as DO4S starting structure. DO3S and DO4S4Me initial geometries were obtained by modifying DO4S.

**Figure 4:**
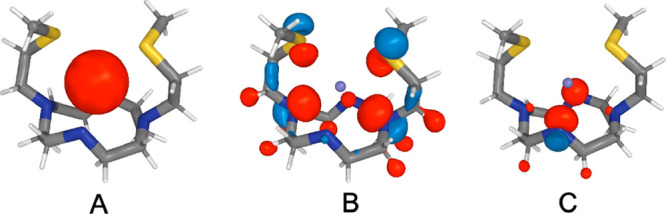
Significant symmetry-adapted fragment orbitals (SFO) for the Ag L^+^ complex formed by DO4S. The extended sulphide side arms from N4 and N10 (nitrogen numbering is shown) have been hidden for the sake of clarity. (A) SFO representing the 5s orbital located on the Ag^+^ center. (B) Ligand SFO involved in the main bonding orbital. (C) Ligand SFO involved in the weaker bonding interaction.

The obtained results are shown in Figure 4. It is well-known that in general Ag^+^ forms linear complexes. However, in the AgL^+^ complexes formed by DO4S, DO4S4Me, and DO3S, the metal d molecular orbitals (MO) energy pattern closely resembles the distinctive order typical of a distorted square planar coordination system, where two pnictogen and two chalcogen atoms act as Lewis bases and each ligand atom behaves like a 2-electron donor system. Furthermore, an in-depth analysis of the correlation diagrams and the evaluation of the metal–ligand overlap integrals show a variation of the interaction strength between the ligands and the Ag^+^ center symmetry-adapted fragment orbitals (SFO). The stronger interaction unravels the nature of the principal bonding force, involving the empty 5s Ag orbital (Figure 4A) and four p orbitals (Figure 4B) belonging to two opposite-side N atoms (i.e., N1 and N7 on the cyclen ring) and to the corresponding S atoms. The fragments’ combination forms the inner valence HOMO–7 (Ag^+^-DO4S, Ag^+^-DO4S4Me), HOMO–8 (Ag^+^-DO3S) bonding and LUMO+2 (Ag^+^-DO4S, Ag^+^-DO3S), LUMO+3 (Ag^+^-DO4S4Me) antibonding pair. HOMO and LUMO have also a smaller contribution formed from the combination of a ligand SFO (Figure 4C) due to the other two opposite N atoms (N4 and N10 on the cyclen ring) and the 5s empty orbital of Ag^+^ (Figure 4A). Due to a poorer overlap and a higher energy gap, the involved interaction is less significant compared to the former one and therefore the arms involving these orbitals do not effectively coordinate the metal center. For these systems, the valence orbitals show no noteworthy combination between metal and ligand but are mainly formed by the almost unperturbed d metal orbitals and few distant orbitals on the ligand pendants. The AgHL^2+^ complex formed by DO4S shows a slightly different bonding mode, because after the insertion of a proton the metal ion slips away from the center of the cyclen ring increasing the distortion of the original square planar coordination. The bonding and antibonding pair are formed by the HOMO–7 and LUMO, respectively. The ligand contribution is similar to the AgL^+^ form (Figure 4B) but with a reduced contribution of both the chalcogens. However, in this case Ag^+^ is closer to the N4 nitrogen and this can contribute significantly through a dumbbell-shaped orbital pointing directly toward the silver atom.

The activation strain analysis (ASA) and the energy decomposition analysis (EDA) have been used to better outline the difference in the bonding nature among the considered ligands in gas-phase (Table 3). The deformation energies Δ*E*_strain_ directly reflect the ligand size: the bigger the ligand is, the higher the strain is. DO3S is more stable in the gas phase because of the reduced strain, but an additional important effect could be delineated upon removal of a (nonmetal-coordinated) sulfanyl pendant arm: the higher electrostatic interaction Δ*V*_elstat_ contributes to increase the overall stabilizing energy Δ*E*_int_. Analogously, the addition of methyl groups in DO4S4Me also contributes to an overstabilization due to more effective electrostatic interactions. Nevertheless, this effect is counterbalanced in the gas phase by a significant increase in the steric repulsion Δ*E*_strain_ as reported above. In all three systems, the orbital interaction term Δ*E*_oi_ does not seem to play a crucial role. The stability order elucidated experimentally in solution was however reversed, likely because the conformational effects characterizing these systems have been neglected in the calculations.

**Table 3:** Activation Strain Analysis (ASA) and Energy Decomposition Analysis (EDA)^a^

ligand	complex	Δ*E*	Δ*E*_strain_	Δ*E*_int_	Δ*E*_Pauli_	Δ*E*_oi_	Δ*V*_elstat_
DO4S	AgL^+^	–98.4	6.7	–105.1	101.6	–82.8	–123.9
DO4S	AgHL^2+^	–19.8	11.7	–31.5	121.4	–93.4	–59.5
DO4S4Me	AgL^+^	–97.4	9.2	–106.6	104.9	–84.5	–127.0
DO3S	AgL^+^	–101.8	5.7	–107.5	105.5	–83.1	–129.9

a For the three AgL^+^ complexes formed by DO4S, DO4S4Me, and DO3S, and for the AgHL^2+^ complex formed by DO4S. All of the energies are in kcal mol^–1^.

Compared to AgL^+^, in AgHL^2+^ a greater Δ*E*_int_ and a less stabilizing electrostatic interaction Δ*V*_elstat_ can be observed. This originates from the more unsymmetrical cyclic scaffold due to the formation of an internal H-bond between NH^+^ and the opposite N. This feature has been found also in the free ligand and has been already discussed in our previous work.^37^ The higher (less stabilizing) Δ*V*_elstat_ is mainly caused by the localized charge on the N10 nitrogen. Strain and interaction contributions for AgHL^2+^ sum up to a generally more unstable protonated form compared to the deprotonated ones. As a result, the p*K*_a_ due to the deprotonation of AgHL^2+^ to form AgL^+^ is relatively small (e.g., for DO4S p*K*_a(AgHL)_ = 4.16 = 21.029 −16.513, see Table 1), and it is much smaller than that due to the deprotonation of the free ligand with the same charge +2 (e.g., for DO4S p*K*_a(H2L)_ = 7.29).^37^

### NMR Characterization in Aqueous Solution

**Figure 5:**
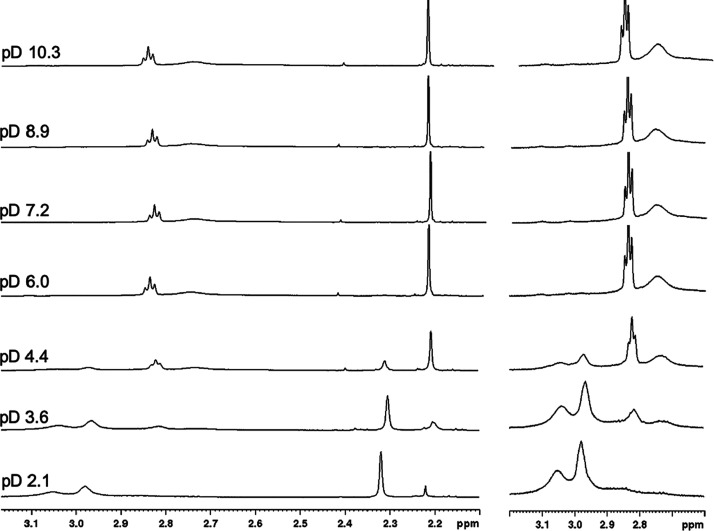
^1^H NMR spectra of solutions containing Ag^+^ and DO4S (600 MHz, 25 °C, D_2_O, *C*_Ag_ = *C*_DOT4S_ = 1.1 × 10^–3^ M) at various pD values. The spectral region in the range of 2.60–3.20 ppm was enlarged for clarity.

The ^1^H NMR spectra of D_2_O solutions containing Ag^+^ and DO4S in the pD range 2–10 are shown in Figure 5; the whole spectral data are summarized in Table S2. The addition of Ag^+^ to the free ligand (spectra of free DO4S were already published in a previous work^37^) caused significant changes in chemical shifts and coupling patterns (Figure S6), thus proving the complex formation at different pD. At pD > 6.0 all spectra are identical: this finding agrees with thermodynamic results according to which only the AgL^+^ complex exists at neutral-to-basic pH. On the basis of the integration values and the bidimensional HMQC spectrum (Figure S7), the singlet at 2.22 ppm and the triplet at 2.84 ppm were assigned to SCH_3_ and SCH_2_, respectively, whereas the very broad singlet centered around at 2.77 ppm was attributed to both ring and arms NCH_2_. Spectra are consistent with the formation of a highly symmetric complex as they exhibit only three resonances, as also observed by Mäcke et al. for the same complex in organic solvent.^38^ This result, combined with the equivalence of all carbon atoms of the side chains (Figure S7), would suggest that all the four sulfur donor atoms are involved in the coordination of Ag^+^. However, according to the DFT calculations herein reported and to the X-ray crystal structure of [AgDO4S]^+^ obtained by Mäcke et al. only two sulfur atoms are simultaneously interacting with the Ag^+^ core and the coordination is completed by the nitrogen atoms of the heterocyclic ring.^38^ Therefore, it is more reasonable to assume that while in the solid state only two sulfurs are effectively bound to the metal ion, all four pendant arms are exchanging fast on the NMR time scale thus becoming chemically equivalent in solution. Accordingly, more conformers of AgL^+^ are exchanging in solution, as also confirmed by both the broadness of the NCH_2_ signal and the exchange cross-peaks (black) in the NOESY spectra (Figure S8).

When the ^1^H NMR spectra at pD > 6.0 are compared with those at lower pD, several differences can be evidenced. This finding can be attributed to the predominance of different complexes in the two conditions, namely AgL^+^ and AgHL^2+^, respectively. As seen in the DFT section, different structural features are expected for these species and these are reflected on the spectra. According to the integration values and the HMQC and NOESY spectra (Figures S9 and S10), the singlet at 2.32 ppm in the spectrum at pD 2.1 was attributed to SCH_3_, while the multiplets at 2.98 and 3.05 ppm were attributed to SCH_2_ and NCH_2_, respectively. The singlet at 2.20 ppm was associated with the SCH_3_ protons of the free ligand. Interestingly, all signals of AgHL^2+^ are broader than those of AgL^+^ so that more conformers and/or slower rates of interconversion are occurring for AgHL^2+^ than for AgL^+^. However, similar upfield-downfield shifts for both complexes with respect to the free ligand are observed (Figures S6 and S11). At pD 3.6 and 4.4 (Figure 5), where AgHL^2+^ and AgL^+^ coexist, the patterns of both complexes can be recognized, indicating that the deprotonation AgHL^2+^ ⇌ AgL^+^ + H^+^ is relatively slow. Also, for the free ligand the deprotonation H_2_L^2+^ ⇌ HL^+^ + H^+^ was slow, and it was attributed to structural changes occurring by the proton loss.^37^

The ^1^H NMR spectra of Ag^+^-DO3S are shown in Figures S12 and S13; signal assignment is summarized in Table S3. The spectra change with pD only in the 5.4–7.8 range: below pD 5.4 and above pD 7.8 no further changes can be evidenced. This behavior agrees with the thermodynamic data according to which two different complexes exist at acidic and at neutral-to-basic conditions, that is, AgHL^2+^ and AgL^+^, respectively. An important difference can be evidenced between the AgL^+^ signals of DO3S and of DO4S: for the former, two different singlets for SCH_3_ protons, at 2.23 and 2.19 ppm, can be observed which implies that the AgL^+^ complex formed by DO3S is asymmetric. In the case of the Cd^2+^-DO3S complexes,^37^ the asymmetry of CdL^2+^ and the coordination of only two among the three sulfanyl pendant arms was demonstrated by the NMR coupling between the SCH_3_ protons and ^111/113^Cd^2+^, whereas for Ag^+^ the coordination of only the two opposite sulfanyl arms (bound to N1 and N7 on the cyclen ring) was demonstrated by DFT structural analysis. It can be assumed that the DO3S arm bound to N4 cannot engage in the metal binding because there is no counter arm on N10, thus resulting chemically different from the other two.

Semiquantitative data can be obtained from the ^1^H NMR spectra by calculating the relative integral between the signals of the complexes and those of the free ligand. For DO4S, the relative amount of AgHL^2+^ and free ligand are 86% and 14%, respectively, at pD 2.1, whereas for DO3S the corresponding percentages are 91% and 9% at pH 3. Taking into account the uncertainty of the NMR integration values and the isotopic and solvent effects, these values are in good agreement with those calculated on the basis of the thermodynamic data of Table 1 (94% and 6% for DO4S and 90% and 10% for DO3S, respectively, at the two given pH).

The ^1^H NMR spectra of solutions containing Ag^+^ and DO3SAm are shown in Figure S14; signal assignment is summarized in Table S4. Similar ^1^H NMR spectra were expected for DO3S and DO3SAm as the two ligands are identical apart from the N-alkylation with the amide group in the latter. This is partly true at pD > 4 but not at more acidic conditions, as for DO3SAm all signals are much more enlarged. This indicates that the AgLH^2+^ complex formed by this ligand has more conformers than that formed by DO3S, and/or that the former experiences slower exchange reactions.

The ^1^H NMR spectra of solutions containing Ag^+^ and DO2A2S are shown in Figure S15; signal assignment is summarized in Table S5 as deduced from HMQC as well (Figure S16). In alkaline solution (pD > 7.8), the spectra are identical as only AgL^–^ exists in these conditions, whereas at lower pD the spectra change because of the presence of AgHL and/or of AgH_2_L^+^. Both ring and side arms NCH_2_ protons give broad multiplets indicating a highly flexible structure as demonstrated by the in-phase correlation peaks (black) in the NOESY spectra (Figure S17). SCH_3_ and SCH_2_ signals are downfield shifted with respect to the free ligand^37^ suggesting the role of the transannular S-donor atoms in the coordination of Ag^+^.

**Figure 6:**
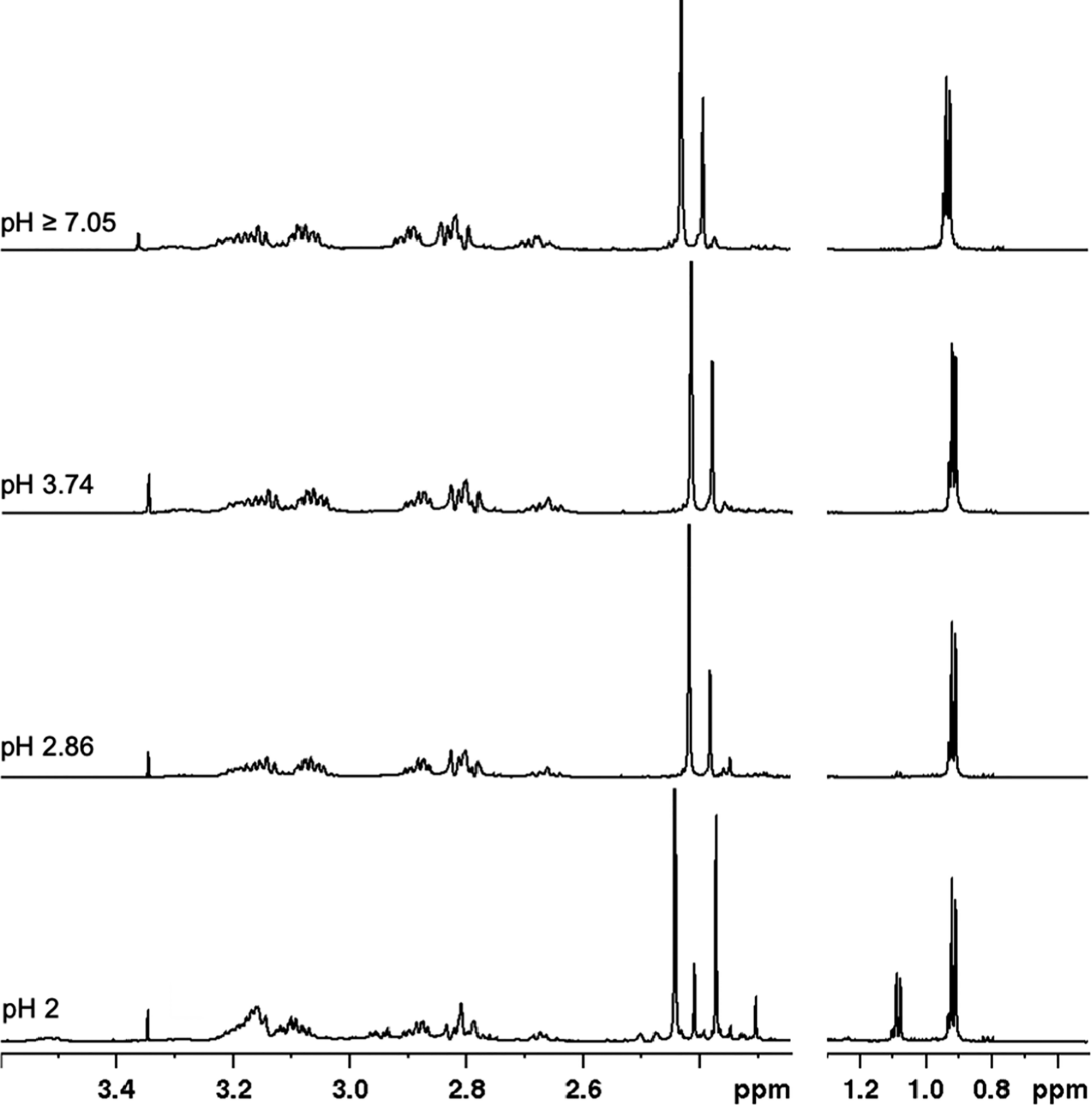
^1^H NMR spectra of solutions containing Ag^+^ and DO4S4Me (600 MHz, 25 °C, H_2_O + 10% D_2_O, *C*_Ag_ = *C*_DO4S4Me_ = 1.1 × 10^–3^ M) at various pH values. The singlet at 3.35 ppm is related to methanol impurity.

The ^1^H NMR spectra of free DO4S4Me are shown in Figures S18, S19, and S20; those of Ag^+^-DO4S4Me are reported in Figure 6. Signal assignment is summarized in Table S6 as deduced also from the COSY spectra (Figures S21 and S22). Clearly, the coordination of Ag^+^ causes significant changes on the spectra (Figure S23); in particular, a large number of narrow signals can be detected in the Ag^+^-DO4S4Me mixture (compare also Figures S24 and S25 with Figures S26 and S27). This feature represents the most evident difference with respect to the spectra obtained for other Ag^+^-ligand solutions (e.g., compare Figure 6 with Figure 5) where few and broader peaks were detected. DO4S4Me forms an asymmetric complex with Ag^+^, and NMR indicates that it is characterized by a slowed-down fluxional interconversion compared to its achiral analogue (Figures S24 and S25). Namely, the chiral methyl groups on the cyclen ring induce the formation of a more rigid complex structure and rise the energetic barrier of interconversion between conformers. Two signals (at around 2.4 ppm, area ratio among 3.5:1 and 3:1) appear for the SCH_3_ protons, and less clearly still two signals with the same ratio appear also for the ring methyl protons (0.9 ppm). This feature might be explained by the formation of two conformers in solution, which are not exchanging on the NMR time scale; the alternative hypothesis, that is, that one of the four sulfur atoms is chemically different from the other three, is also possible, however it is not supported by DFT according to which the chalcogen atoms are equivalent two-by-two. At pH 2, the appearance of a new ring CH_3_ signal at 1.08 ppm and of (at least) one additional SCH_3_ peak at 2.37 ppm, evidence that a different complex coexists, which is identified as AgHL^2+^ according to the thermodynamic data.

### Radiolabeling Experiments

The compounds forming the most stable complexes with Ag^+^, that is, DO4S and DO4S4Me, were used to investigated their capability of labeling [^111^Ag]Ag^+^ after the purification process. Because of the low amount of ^111^Ag available, only one data point was collected for each experiment parameter. For this reason, the results obtained should be consider preliminary and a more thorough evaluation should be conducted in future radiolabeling studies.

Results of radiolabeling are summarized in Table 4. For DO4S at pH 4 with a 2 × 10^–5^ M ligand concentration, a quantitative yield both at room temperature (RT) and at 50 °C was achieved in 5 min. The incorporation of [^111^Ag]Ag^+^ remains quantitative at pH 7, whereas it becomes lower than 75% at pH 2. This behavior was expected from thermodynamic data according to which some free metal ions exist at very acidic pH values. The radionuclide incorporation by DO4S4Me in the same conditions was similar, even if the temperature appears to be a relevant parameter as at RT a lower yield of 76% was obtained at pH 4. Concentration-dependent radiolabeling at 50 °C and pH 4 indicated that for both ligands the complexation was quantitative in 5 min with a minimum concentration of 2 × 10^–5^ M (20 nmol). Efficiency became lower when the ligand concentration was reduced. Although thermodynamic data predicted a stronger complex formation for DO4S4Me than for DO4S, the labeling experiments demonstrated the former to be slight less efficient than the latter. Indeed, according to the temperature effect evidenced on the complex formation for DO4S4Me, this ligand might react with Ag^+^ more slowly than DO4S, so that the addition of a rigid chiral backbone onto the DO4S structure with the intention of increasing its stability may also hamper the labeling kinetics at the lowest concentrations.

**Table 4:** [^111^Ag]Ag^+^ Radiochemical Yields (in %) for DO4S and DO4S4Me^a^

ligand	ligand molarity [M]	temperature [°C]	pH	yields [%]
DO4S	1 × 10^–6^	50	4	85
	1 × 10^–5^	50	4	87
	2 × 10^–5^	50	4	100
	2 × 10^–5^	50	2	75
	2 × 10^–5^	50	7	100
	2 × 10^–5^	RT	4	100
DO4S4Me	1 × 10^–6^	50	4	53
	1 × 10^–5^	50	4	92
	2 × 10^–5^	50	4	100
	2 × 10^–5^	RT	4	76

a All yields are given within the experimental uncertainties of the cyclone device of 5% and refer to a labeling time of 5 min and to a ∼ 1 MBq ^111^Ag activity.

The stability of the [^111^Ag]Ag^+^ complexes formed by the two ligands was investigated versus time after incubation in suitable media (Table 5). In saline phosphate buffer, the stability was 94% and 88% over 48 h for DO4S and DO4S4Me, respectively, thus demonstrating high stability at the employed conditions. Clearly, longer time evaluations, also in more relevant biological media (serum, blood), will be performed in future work.

**Table 5:** Stability of [^111^Ag]Ag^+^-Labelled Chelators at RT^a^

	PBS			5 × Zn^2+^			2 × Cu^2+^	2 × Cd^2+^
complex	2 h	24 h	48 h	0 h	24 h	48 h		
[^111^Ag]Ag^+^-DO4S	100	94	94	100	100	100	92	95
[^111^Ag]Ag^+^-DO4S4Me	90	88	88	100	100	100	87	96

a In phosphate saline buffer (PBS, pH 7.4) or with a 5-fold molar excess of Zn^2+^ (5 × Zn^2+^), and labeling efficiency in the presence of a 2-fold molar excess of Cu^2+^ or of Cd^2+^ (2 × Cu^2+^, 2 × Cd^2+^). Values are represented as % intact complex and are given within the experimental uncertainties of the cyclone device of 5%.

A check of metal-transmetalation possibly occurring *in vivo* was performed with Zn^2+^, and very encouraging results were obtained, as both [^111^Ag]Ag^+^ complexes were completely intact (100%) over time in the presence of a 5-fold Zn^2+^ excess. Furthermore, in competition experiments an almost complete labeling was obtained when [^111^Ag]Ag^+^ and ligand were mixed in the presence of a 2-fold molar excess of Cu^2+^ or of Cd^2+^. Copper was chosen because of its physiological relevance, whereas Cd^2+^ was shown to form very stable complexes with cyclen sulfanyl derivatives^37^ and it, therefore, represented a valuable test check for metal competition studies.

## Conclusions

In this work, several sulfide-containing derivatives of cyclen have been considered as Ag^+^ chelators. Potentiometric and spectrophotometric results showed that these compounds formed a very stable AgL complex at physiological pH, but complex stability was remarkably high also in very acidic solutions where protonated species (e.g., AgHL) predominated. Compounds bearing four sulfide arms (DO4S and DO4S4Me) were demonstrated to form the most stable complexes. The complexes formed by compounds with three sulfide chains, that is, DO3S and DO3SAm, were however not much weaker: this finding was explained by DFT calculations, NMR measurements, and the available X-ray structure of DO4S,^38^ which indicated that only two among the four sulfur atoms can simultaneously coordinate Ag^+^. The highest stability displayed by the compounds bearing four sulfide arms can thus be attributed to statistical effects.

The formation of a distorted tetrahedral structure around Ag^+^ was demonstrated through DFT calculations. Among the eight possible coordinating groups of DO4S, only the two opposite N atoms (i.e., N1 and N7 on the cyclen ring) and the corresponding sulfide arms were bound to Ag^+^. A weaker coordination by the remaining two nitrogens was also computed to occur. Detailed energy calculations indicated that the AgL^+^ complexes of DO4S, DO3S, and DO4S4Me have similar properties, whereas the AgHL^2+^ complex of DO4S is less stable due to higher distortion.

The high thermodynamic stability obtained with natural Ag^+^ was confirmed by preliminary experiments performed with [^111^Ag]Ag^+^. Radiolabeling data showed that the metal ion was rapidly and efficiently bound by DO4S at various pH and also at room temperature. DO4S4Me, on the other hand, gave quantitative radiolabeling only at a higher temperature, suggesting that the addition of a rigid chiral backbone onto the DO4S structure with the intention of increasing its stability may also hamper the labeling kinetics. Both ligands were indeed effective in preventing transmetalation reactions with Zn^2+^, and they formed selectively a complex with Ag^+^ in the presence of Cu^2+^ and Cd^2+^.

All these results strongly suggest that the proposed class of ligands, including sulfide side arms on a cyclen backbone, are very promising to be used as bifunctional chelators in nuclear medicine when ^111^Ag (or another soft metal ion) is used as a theranostic radioisotope. As well, the very high affinity of these ligands toward Ag^+^ may suggest their use for the recovery or the recycling of this metal ion at environmental conditions. DOTA and its analogues, the most used metal chelators in environmental and clinical chemistry, are devoted to strongly complex only hard or borderline ions whereas the sulfanyl derivatives were actually shown to form much stronger complexes with Ag^+^. In this connection, the latter can be proposed as a complementary class of ligands which are able to complex those ions which cannot be as strongly bound by DOTA-like compounds.

## Supporting Information Available

Table of the Cartesian coordinates of equilibrium structures computed at COSMO-OPBE/TZ2P//OPBE/TZP, DZP, tables of the ^1^H NMR signals of Ag^+^-ligand complexes, figures of the ^1^H NMR spectra (mono- and bidimensional spectra), and UV–vis spectra of Ag^+^-ligand complexes (PDF)
