# Supplementary material for: Highly Stable Silver(I) Complexes with Cyclen-Based Ligands Bearing Sulfide Arms: A Step Toward Silver-111 Labeled Radiopharmaceuticals
Source: Inorg Chem. 2020 Jul 13;59(15):10907–19. doi: 10.1021/acs.inorgchem.0c01405 (PMC8009516; doi:10.1021/acs.inorgchem.0c01405)
Supplement: Supplementary file 1 — ic0c01405_si_001.pdf [file ic0c01405_si_001.pdf]

Supporting Information

Highly Stable Silver(I) Complexes with Cyclen-Based Ligands Bearing Sulphide Arms: a Step Towards Silver-111 Labeled Radiopharmaceuticals

Marianna Tosato,[1] Mattia Asti,[2] Marco Dalla Tiezza,[1] Laura Orian,[1] Daniel Häussinger,[3] Raphael Vogel,[3] Ulli Köster,[4] Mikael Jensen,[5] Alberto Andrighetto,[6] Paolo Pastore,[1] Valerio Di Marco[1,*]

[1] Department of Chemical Sciences, University of Padova, via Marzolo 1, 35131 Padova, Italy.

[2] Radiopharmaceutical Chemistry Section, Nuclear Medicine Unit, AUSL-IRCCS di Reggio Emilia, Viale Risorgimento 80, 42122 Reggio Emilia, Italy.

[3] Department of Chemistry, University of Basel, St. Johannsring 19, 4056, Basel, Switzerland.

[4] Institut Laue-Langevin, 71 avenue des Martyrs CS 20156, 38042 Grenoble Cedex 9, France.

[5] The Hevesy Laboratory, Dept. Health Technology, Technical University of Denmark (DTU), Frederiksborgvej 399, 4000, Roskilde, Denmark.

[6] Italian Institute of Nuclear Physics, Legnaro National Laboratories, Viale dell'Università 2, 35020 Legnaro (Padova), Italy.

*Corresponding Author: valerio.dimarco@unipd.it

Table S1. Cartesian coordinates (in Å) of equilibrium structures computed at COSMO-OPBE/TZ2P//OPBE/TZP, DZP.

DO4S

S	9.711400000	-2.566300000	-6.554500000
S	4.917300000	-4.710000000	-8.849300000
S	2.729900000	-1.771100000	-4.062800000
S	7.532700000	0.168000000	-1.668200000
N	7.995900000	-0.071800000	-8.247000000
N	5.059800000	-0.618900000	-8.182300000
N	3.821900000	1.019700000	-5.789100000
N	6.803600000	1.198500000	-5.618200000
C	7.213900000	-0.318000000	-9.446400000
H	7.375800000	-1.363600000	-9.737000000
H	7.572800000	0.288400000	-10.309600000
C	5.711700000	-0.066300000	-9.341100000
H	5.528700000	1.016700000	-9.337000000
H	5.277200000	-0.413400000	-10.306500000
C	3.716100000	-0.108700000	-8.001900000
H	3.141000000	-0.850100000	-7.435900000
H	3.185500000	0.034500000	-8.965900000
C	3.692300000	1.209800000	-7.227400000
H	4.528200000	1.820000000	-7.575300000
H	2.772100000	1.777300000	-7.480500000
C	4.511300000	2.115200000	-5.129300000
H	4.489000000	1.916900000	-4.050500000
H	3.977700000	3.084300000	-5.263700000
C	5.957800000	2.361900000	-5.551200000
H	5.966600000	2.833600000	-6.542800000
H	6.341100000	3.154500000	-4.868400000
C	8.059600000	1.459400000	-6.291300000
H	8.796900000	0.732700000	-5.931900000
H	8.464900000	2.465300000	-6.056000000
C	7.948900000	1.316500000	-7.809700000
H	6.995300000	1.747400000	-8.121300000
H	8.735900000	1.924900000	-8.302700000
C	9.370600000	-0.497200000	-8.455100000
H	10.014500000	0.048600000	-7.756000000
H	9.725100000	-0.218600000	-9.473500000
C	9.614000000	-1.991000000	-8.263100000
H	8.813400000	-2.584600000	-8.720700000
H	10.542900000	-2.282100000	-8.776800000
C	11.413100000	-2.140100000	-6.167900000
H	12.118500000	-2.676900000	-6.815400000
H	11.585400000	-2.458300000	-5.132800000
H	11.602100000	-1.061600000	-6.227500000
C	5.217400000	-2.041900000	-7.981400000
H	4.799400000	-2.268800000	-6.994400000
H	6.288000000	-2.263000000	-7.910600000
C	4.578200000	-2.946600000	-9.039100000
H	3.489600000	-2.796200000	-9.093200000
H	4.976600000	-2.717100000	-10.037500000
C	3.950500000	-5.103900000	-7.386300000
H	4.343700000	-4.630500000	-6.480900000
H	4.013800000	-6.191800000	-7.262500000
H	2.896100000	-4.834200000	-7.524700000
C	2.516000000	0.819200000	-5.182200000
H	1.872500000	0.301000000	-5.902500000
H	2.016900000	1.793500000	-4.976400000
C	2.526900000	0.013900000	-3.887000000
H	3.344000000	0.332400000	-3.228300000
H	1.595900000	0.201200000	-3.330800000
C	1.043500000	-2.251800000	-4.452500000
H	1.052500000	-3.341100000	-4.577400000
H	0.682600000	-1.806100000	-5.387100000
H	0.355600000	-2.004400000	-3.633500000
C	6.901000000	0.412700000	-4.408500000
H	7.440500000	-0.504100000	-4.671400000
H	5.892400000	0.095700000	-4.121400000
C	7.581100000	1.093600000	-3.217300000
H	8.625200000	1.354700000	-3.445300000
H	7.070200000	2.033600000	-2.965500000
C	8.630200000	-1.207300000	-2.034200000
H	8.753400000	-1.757400000	-1.093600000
H	9.616600000	-0.851600000	-2.356400000
H	8.215100000	-1.891100000	-2.781700000

Ag^{+}-DO4S

Ag	6.083100000	-0.967500000	-6.100900000
S	7.875200000	-3.063100000	-6.176500000
S	3.796000000	-4.182100000	-10.647800000
S	4.673900000	-2.037500000	-4.016100000
S	8.309100000	2.215700000	-1.497100000
N	7.783800000	-0.255100000	-7.907300000
N	4.812500000	-0.859400000	-8.366500000
N	4.101300000	0.676500000	-5.792600000
N	7.065800000	1.321000000	-5.359300000
C	7.140400000	-0.428700000	-9.210700000
H	7.258800000	-1.471900000	-9.517400000
H	7.651100000	0.170700000	-9.990000000
C	5.661900000	-0.065200000	-9.248200000
H	5.544000000	0.985600000	-8.967300000
H	5.339300000	-0.118600000	-10.303700000
C	3.538100000	-0.198100000	-8.097300000
H	2.855600000	-0.957100000	-7.696900000
H	3.060000000	0.181300000	-9.020200000
C	3.608100000	0.982100000	-7.134600000
H	4.250000000	1.758100000	-7.562200000
H	2.595600000	1.430800000	-7.094100000
C	4.624700000	1.881000000	-5.147500000
H	4.644100000	1.713100000	-4.066800000
H	3.946800000	2.743500000	-5.302400000
C	6.013700000	2.300600000	-5.610400000
H	5.988700000	2.495300000	-6.686700000
H	6.230500000	3.279600000	-5.146100000
C	8.238500000	1.553600000	-6.198600000
H	9.071500000	0.994300000	-5.756400000
H	8.551800000	2.614700000	-6.195800000
C	8.078800000	1.156500000	-7.662100000
H	7.279200000	1.752500000	-8.111900000
H	9.004200000	1.465600000	-8.187400000
C	9.005100000	-1.049900000	-7.819000000
H	9.593600000	-0.679800000	-6.973300000
H	9.636700000	-0.911200000	-8.720500000
C	8.794200000	-2.546200000	-7.637500000
H	8.252700000	-2.990000000	-8.482500000
H	9.775700000	-3.042400000	-7.623900000
C	9.129100000	-2.934300000	-4.895700000
H	9.943800000	-3.642000000	-5.092000000
H	8.643000000	-3.220800000	-3.955900000
H	9.526600000	-1.921400000	-4.791100000
C	4.615700000	-2.238800000	-8.834300000
H	4.097300000	-2.775000000	-8.032000000
H	5.594300000	-2.720100000	-8.928800000
C	3.855500000	-2.450600000	-10.144400000
H	2.831800000	-2.055200000	-10.101700000
H	4.357000000	-1.951100000	-10.983600000
C	2.524700000	-4.833600000	-9.558900000
H	2.833200000	-4.865700000	-8.508500000

H	2.348700000	-5.864500000	-9.889200000
H	1.584700000	-4.279100000	-9.668400000
C	3.047400000	0.069100000	-4.987000000
H	2.495300000	-0.634300000	-5.618700000
H	2.311100000	0.828900000	-4.653100000
C	3.527100000	-0.673700000	-3.748200000
H	4.041500000	-0.007800000	-3.043900000
H	2.651900000	-1.058200000	-3.204300000
C	3.562700000	-3.343900000	-4.552200000
H	4.190100000	-4.216100000	-4.769400000
H	3.003100000	-3.080200000	-5.453300000
H	2.872200000	-3.608300000	-3.742100000
C	7.430800000	1.212900000	-3.939700000
H	8.101900000	0.352000000	-3.848500000
H	6.534800000	0.954200000	-3.366800000
C	8.084400000	2.424700000	-3.274400000
H	9.050000000	2.680400000	-3.731100000
H	7.447500000	3.315700000	-3.351800000
C	9.709900000	1.092900000	-1.437800000
H	9.986500000	1.015800000	-0.379300000
H	10.570200000	1.501100000	-1.982300000
H	9.469100000	0.086200000	-1.796200000

DO4S (AgHL^{2+})

S	9.491300000	-2.632400000	-6.673500000
S	5.267400000	-4.551100000	-8.550900000
S	2.714600000	-1.755700000	-4.313900000
S	7.251300000	-0.158000000	-1.989900000
N	8.056000000	-0.047100000	-8.364800000
N	5.175600000	-0.450400000	-7.962600000
N	3.690100000	1.136800000	-5.789500000
N	6.634300000	1.081100000	-5.908900000
C	7.197200000	-0.258800000	-9.504100000
H	7.287700000	-1.308300000	-9.808200000
H	7.484800000	0.339200000	-10.397100000
C	5.733700000	0.059100000	-9.249500000
H	5.555700000	1.139500000	-9.245300000
H	5.134100000	-0.351400000	-10.070200000
C	3.719400000	-0.096000000	-7.876700000
H	3.238600000	-0.858800000	-7.256200000
H	3.298900000	-0.137100000	-8.888600000
C	3.498900000	1.253600000	-7.221700000
H	4.182100000	2.000200000	-7.641000000
H	2.488100000	1.609700000	-7.490600000
C	4.476900000	2.203200000	-5.201900000
H	4.523800000	2.011800000	-4.123500000
H	3.996600000	3.198900000	-5.311300000
C	5.892400000	2.335000000	-5.745600000
H	5.875600000	2.834900000	-6.724200000
H	6.421700000	3.039900000	-5.078900000
C	7.994100000	1.387800000	-6.389600000
H	8.676200000	0.630400000	-5.988900000
H	8.337500000	2.368900000	-6.021200000
C	8.098300000	1.337700000	-7.903000000
H	7.283400000	1.917200000	-8.351700000
H	9.022100000	1.846900000	-8.230300000
C	9.399000000	-0.574300000	-8.604400000
H	10.089000000	-0.061400000	-7.925800000
H	9.739700000	-0.329300000	-9.632000000
C	9.556300000	-2.073500000	-8.387700000
H	8.788700000	-2.649200000	-8.920800000
H	10.515600000	-2.393200000	-8.821600000
C	11.179100000	-2.305100000	-6.153100000
H	11.893500000	-2.894000000	-6.741100000
H	11.247300000	-2.629100000	-5.108200000
H	11.441400000	-1.242000000	-6.198700000
C	5.417200000	-1.914200000	-7.733200000
H	5.042500000	-2.108500000	-6.723500000
H	6.500500000	-2.047300000	-7.723100000
C	4.752700000	-2.834100000	-8.740100000
H	3.656900000	-2.774600000	-8.695900000
H	5.049300000	-2.589500000	-9.768300000
C	4.381500000	-5.044700000	-7.069200000
H	4.750100000	-4.551200000	-6.164500000
H	4.564600000	-6.121200000	-6.966600000
H	3.301900000	-4.893300000	-7.182900000
C	2.433200000	0.939900000	-5.069600000
H	1.686300000	0.540200000	-5.765300000
H	2.030900000	1.910000000	-4.713400000
C	2.534200000	-0.011500000	-3.885200000
H	3.390800000	0.233500000	-3.244900000
H	1.642500000	0.098800000	-3.250900000
C	0.993100000	-2.225700000	-4.527000000
H	0.992600000	-3.288700000	-4.795000000
H	0.493600000	-1.667900000	-5.327500000
H	0.438000000	-2.111200000	-3.588200000
C	6.671300000	0.267300000	-4.680100000
H	7.203000000	-0.655300000	-4.939300000
H	5.643300000	-0.018000000	-4.439800000
C	7.322100000	0.898500000	-3.451200000
H	8.367400000	1.182100000	-3.634900000
H	6.798200000	1.813500000	-3.144900000
C	8.612600000	-1.288200000	-2.297200000
H	8.668800000	-1.934900000	-1.413200000
H	9.562700000	-0.747500000	-2.384800000
H	8.448900000	-1.922000000	-3.174000000
H	5.702200000	0.067000000	-7.166400000

Ag^{+}-DO4S (AgHL^{2+})

Ag	6.415800000	-1.097200000	-5.375100000
S	7.827100000	-3.113900000	-6.075500000
S	3.767800000	-4.180600000	-10.596900000
S	4.662300000	-1.969500000	-3.727200000
S	8.419700000	2.708000000	-1.587200000
N	7.688400000	-0.311600000	-7.904300000
N	4.778000000	-0.764700000	-8.481000000
N	4.083600000	0.516100000	-5.868300000
N	7.093900000	1.195800000	-5.231100000
C	7.143200000	-0.401600000	-9.258300000
H	7.255200000	-1.428500000	-9.620800000
H	7.704600000	0.225400000	-9.978200000
C	5.688900000	0.019100000	-9.370700000
H	5.558100000	1.070800000	-9.103500000
H	5.341600000	-0.095900000	-10.402600000
C	3.476000000	-0.070600000	-8.228100000
H	2.754300000	-0.842600000	-7.946100000
H	3.132000000	0.376400000	-9.168100000
C	3.587300000	0.999100000	-7.156800000
H	4.244700000	1.799900000	-7.508600000
H	2.584500000	1.460700000	-7.071700000
C	4.617500000	1.647300000	-5.092200000
H	4.668600000	1.349500000	-4.043100000
H	3.925700000	2.510000000	-5.126100000
C	5.992400000	2.127600000	-5.523000000
H	5.993500000	2.338100000	-6.595200000
H	6.161900000	3.102600000	-5.040800000
C	8.233700000	1.433200000	-6.130100000
H	9.086100000	0.876000000	-5.723400000
H	8.535800000	2.494900000	-6.118300000
C	8.029000000	1.083700000	-7.597500000
H	7.252000000	1.729200000	-8.018300000
H	8.958700000	1.380500000	-8.120400000
C	8.894200000	-1.143400000	-7.798500000
H	9.482100000	-0.786900000	-6.947800000

H	9.538800000	-1.016600000	-8.690200000
C	8.639400000	-2.627400000	-7.613400000
H	8.026000000	-3.055400000	-8.416200000
H	9.597300000	-3.164900000	-7.663600000
C	9.216600000	-3.215300000	-4.936100000
H	9.875500000	-4.035100000	-5.245300000
H	8.799500000	-3.462800000	-3.953300000
H	9.776900000	-2.279300000	-4.865100000
C	4.586900000	-2.205900000	-8.910300000
H	4.032700000	-2.680200000	-8.094500000
H	5.578100000	-2.663100000	-8.939200000
C	3.878300000	-2.415600000	-10.234500000
H	2.875900000	-1.970100000	-10.257500000
H	4.448100000	-1.992500000	-11.071700000
C	2.247700000	-4.632000000	-9.756100000
H	2.313000000	-4.554700000	-8.665600000
H	2.086400000	-5.688400000	-10.005100000
H	1.394800000	-4.063100000	-10.143600000
C	2.985400000	-0.138400000	-5.145100000
H	2.564500000	-0.916900000	-5.791000000
H	2.165600000	0.580900000	-4.948000000
C	3.323200000	-0.766000000	-3.801300000
H	3.575800000	-0.014500000	-3.042600000
H	2.415000000	-1.256500000	-3.421200000
C	3.874500000	-3.451500000	-4.371100000
H	4.635400000	-4.239900000	-4.367700000
H	3.494100000	-3.326600000	-5.387300000
H	3.065400000	-3.753000000	-3.695200000
C	7.538700000	1.316700000	-3.811800000
H	8.205400000	0.469200000	-3.611200000
H	6.661300000	1.178800000	-3.170600000
C	8.244800000	2.601700000	-3.379100000
H	9.233600000	2.712700000	-3.842700000
H	7.664700000	3.496600000	-3.639400000
C	9.817800000	1.622400000	-1.284700000
H	10.053300000	1.740400000	-0.219800000
H	10.698000000	1.936400000	-1.858100000
H	9.591700000	0.565100000	-1.460500000
H	5.258100000	-0.792400000	-7.557400000

DO3S

S	9.143500000	-3.089700000	-6.575300000
S	4.529300000	-4.479300000	-9.692400000
S	2.741900000	-2.292000000	-4.441500000
N	7.932900000	-0.306600000	-8.176000000
N	4.936300000	-0.600800000	-8.293100000
N	3.786800000	0.717100000	-5.699000000
N	6.712500000	0.718500000	-5.533800000
C	7.173600000	-0.304200000	-9.412400000
H	7.243900000	-1.310900000	-9.844100000
H	7.618300000	0.375500000	-10.175600000
C	5.698900000	0.081200000	-9.305800000
H	5.624400000	1.158000000	-9.104900000
H	5.283000000	-0.037700000	-10.333000000
C	3.631600000	-0.015400000	-8.072400000
H	2.973100000	-0.793500000	-7.669900000
H	3.159800000	0.337700000	-9.013400000
C	3.660200000	1.149800000	-7.082300000
H	4.516000000	1.781500000	-7.329500000
H	2.760000000	1.783000000	-7.232300000
C	4.521600000	1.655500000	-4.868700000
H	4.532400000	1.252000000	-3.846800000
H	4.012800000	2.644000000	-4.800500000
C	5.966200000	1.918100000	-5.277900000
H	5.987000000	2.538500000	-6.185000000
H	6.402800000	2.565100000	-4.482600000
C	8.049100000	0.936800000	-6.038200000
H	8.671100000	0.088300000	-5.727900000
H	8.522500000	1.855500000	-5.632200000
C	8.059400000	1.007600000	-7.565000000
H	7.215400000	1.627100000	-7.880200000
H	8.970500000	1.535900000	-7.915200000
C	9.234900000	-0.918100000	-8.377600000
H	9.930900000	-0.512600000	-7.634100000
H	9.660100000	-0.645200000	-9.369800000
C	9.244800000	-2.438300000	-8.257400000
H	8.398200000	-2.882600000	-8.794500000
H	10.154000000	-2.841800000	-8.728900000
C	10.870600000	-2.980400000	-6.092000000
H	11.507700000	-3.607900000	-6.728700000
H	10.931400000	-3.358700000	-5.064400000
H	11.244100000	-1.949300000	-6.095700000
C	4.967000000	-2.043700000	-8.333300000
H	4.491100000	-2.395000000	-7.411000000
H	6.010800000	-2.368400000	-8.271000000
C	4.300000000	-2.694700000	-9.548400000
H	3.224200000	-2.466800000	-9.587000000
H	4.735900000	-2.309400000	-10.481000000
C	3.581000000	-5.081300000	-8.289200000
H	4.037500000	-4.818500000	-7.329400000
H	3.566900000	-6.174300000	-8.377300000
H	2.545800000	-4.719900000	-8.326100000
C	2.486800000	0.442800000	-5.116200000
H	1.825000000	0.057500000	-5.900500000
H	2.005300000	1.375800000	-4.741800000
C	2.507500000	-0.564900000	-3.971900000
H	3.321000000	-0.343100000	-3.270100000
H	1.574500000	-0.486500000	-3.393900000
C	1.049800000	-2.753200000	-4.829800000
H	1.077800000	-3.804200000	-5.141100000
H	0.634200000	-2.165100000	-5.656900000
H	0.397200000	-2.670700000	-3.951000000
H	6.718200000	0.112500000	-4.717000000

Ag^+-DO3S

Ag	6.095700000	-0.989400000	-6.081700000
S	7.857600000	-3.016000000	-6.154800000
S	3.830200000	-4.164400000	-10.658400000
S	4.656800000	-2.040300000	-3.985700000
N	7.819800000	-0.259200000	-7.951000000
N	4.825900000	-0.853500000	-8.349000000
N	4.084500000	0.668900000	-5.776500000
N	7.008700000	1.256900000	-5.468300000
C	7.142900000	-0.416200000	-9.236800000
H	7.249800000	-1.456600000	-9.558200000
H	7.632600000	0.188900000	-10.025300000
C	5.665100000	-0.044600000	-9.229200000
H	5.563400000	0.999000000	-8.916500000
H	5.316200000	-0.067200000	-10.277700000
C	3.541900000	-0.207900000	-8.081100000
H	2.869500000	-0.977000000	-7.682500000
H	3.061300000	0.165100000	-9.005200000
C	3.588300000	0.972500000	-7.116500000
H	4.214500000	1.761500000	-7.542700000
H	2.567300000	1.401500000	-7.078000000
C	4.631000000	1.861800000	-5.133100000
H	4.729000000	1.657500000	-4.060300000
H	3.941200000	2.724200000	-5.220400000
C	5.994900000	2.286600000	-5.655100000
H	5.933400000	2.517500000	-6.722800000
H	6.265100000	3.236400000	-5.157100000
C	8.242900000	1.501600000	-6.208600000
H	9.032900000	0.909100000	-5.730900000
H	8.568400000	2.557100000	-6.148500000

C	8.143500000	1.140200000	-7.686300000
H	7.374200000	1.764100000	-8.152400000
H	9.094200000	1.434600000	-8.172400000
C	9.018500000	-1.084100000	-7.873100000
H	9.639800000	-0.709700000	-7.052600000
H	9.633700000	-0.990100000	-8.791700000
C	8.767000000	-2.567300000	-7.644100000
H	8.193500000	-3.017400000	-8.464500000
H	9.732200000	-3.094700000	-7.632900000
C	9.107000000	-2.803800000	-4.880900000
H	9.950100000	-3.481000000	-5.063300000
H	8.633500000	-3.091600000	-3.935200000
H	9.459700000	-1.772300000	-4.796300000
C	4.637600000	-2.230500000	-8.829400000
H	4.114500000	-2.774000000	-8.034900000
H	5.618200000	-2.708200000	-8.919400000
C	3.888800000	-2.435300000	-10.147200000
H	2.865500000	-2.038300000	-10.112500000
H	4.399200000	-1.933500000	-10.979700000
C	2.544500000	-4.817100000	-9.587400000
H	2.840400000	-4.854300000	-8.533400000
H	2.370000000	-5.846200000	-9.924100000
H	1.607300000	-4.259800000	-9.705800000
C	3.034200000	0.069100000	-4.962600000
H	2.476800000	-0.635000000	-5.588900000
H	2.301700000	0.832200000	-4.626800000
C	3.516800000	-0.670600000	-3.722700000
H	4.037400000	-0.002600000	-3.024200000
H	2.641900000	-1.047500000	-3.173300000
C	3.541000000	-3.343500000	-4.520500000
H	4.164600000	-4.223000000	-4.718700000
H	2.996800000	-3.086400000	-5.433000000
H	2.837100000	-3.593200000	-3.717300000
H	7.242400000	1.220700000	-4.474700000

DO4SMe

S	9.778900000	-2.438900000	-6.582500000
S	5.143700000	-4.762600000	-8.180100000
S	2.592500000	-1.604900000	-4.198900000
S	7.418900000	-0.420800000	-1.884200000
N	8.002200000	0.010500000	-8.316600000
N	5.105200000	-0.614500000	-8.326300000
N	3.817500000	1.160600000	-5.901000000
N	6.761900000	1.322700000	-5.596700000
C	7.265700000	-0.273400000	-9.536200000
H	7.464700000	-1.320300000	-9.802400000
H	7.638500000	0.316700000	-10.400900000
C	5.743900000	-0.076500000	-9.505800000
H	5.556700000	1.008800000	-9.467100000
C	3.790200000	-0.085900000	-8.045000000
H	3.288800000	-0.782900000	-7.365400000
H	3.156900000	-0.024300000	-8.948800000
C	3.830000000	1.300300000	-7.364400000
H	4.817100000	1.704100000	-7.594600000
C	4.473900000	2.256900000	-5.210300000
H	4.397200000	2.057400000	-4.133100000
H	3.947200000	3.224100000	-5.359200000
C	5.951300000	2.518700000	-5.536000000
H	5.990700000	2.945400000	-6.551100000
C	8.010600000	1.466800000	-6.311200000
H	8.666300000	0.645200000	-6.004300000
H	8.541100000	2.401000000	-6.051600000
C	7.843200000	1.393600000	-7.844600000
H	6.795000000	1.631600000	-8.032100000
C	9.393700000	-0.374300000	-8.484800000
H	10.005300000	0.179600000	-7.765000000
H	9.779700000	-0.094100000	-9.488900000
C	9.669000000	-1.864200000	-8.289000000
H	8.882400000	-2.477200000	-8.745000000
H	10.604200000	-2.135900000	-8.802200000
C	11.456600000	-1.938300000	-6.181000000
H	12.188400000	-2.428800000	-6.836000000
H	11.641800000	-2.268200000	-5.151900000
H	11.595100000	-0.851100000	-6.219400000
C	5.372100000	-1.967100000	-7.909900000
H	5.112900000	-2.027000000	-6.846500000
H	6.451100000	-2.139200000	-7.959400000
C	4.648500000	-3.092900000	-8.661600000
H	3.555800000	-3.002000000	-8.570500000
H	4.878600000	-3.056900000	-9.732300000
C	4.372600000	-4.926800000	-6.565200000
H	4.809800000	-4.258400000	-5.817100000
H	4.546900000	-5.962100000	-6.248000000
H	3.290200000	-4.762100000	-6.626000000
C	2.479900000	0.975500000	-5.363700000
H	1.869200000	0.448300000	-6.104300000
H	1.969400000	1.946800000	-5.185200000
C	2.422900000	0.185400000	-4.057900000
H	3.219100000	0.494900000	-3.370300000
H	1.474500000	0.399700000	-3.541800000
C	0.917800000	-2.050000000	-4.672500000
H	0.900900000	-3.142100000	-4.767000000
H	0.622900000	-1.620800000	-5.637600000
H	0.192900000	-1.760600000	-3.900600000
C	6.746700000	0.385100000	-4.501900000
H	7.079800000	-0.577900000	-4.906900000
H	5.709700000	0.231400000	-4.189800000
C	7.598100000	0.724400000	-3.270400000
H	8.664100000	0.808100000	-3.528700000
H	7.301200000	1.689200000	-2.844000000
C	8.281600000	-1.876400000	-2.489800000
H	8.311200000	-2.583100000	-1.651500000
H	9.312400000	-1.634700000	-2.774400000
H	7.766900000	-2.356100000	-3.327900000
C	2.796000000	2.265000000	-7.947800000
H	3.016400000	2.447600000	-9.007900000
H	1.773100000	1.871700000	-7.893700000
H	2.811400000	3.235500000	-7.436700000
C	6.430400000	3.656600000	-4.614000000
H	6.221000000	3.448300000	-3.558900000
H	7.506900000	3.827100000	-4.721900000
H	5.921100000	4.596100000	-4.870000000
C	8.684300000	2.443900000	-8.572700000
H	9.751200000	2.376200000	-8.326100000
H	8.584200000	2.360800000	-9.662000000
H	8.349800000	3.450000000	-8.287000000
C	5.193200000	-0.518000000	-10.875800000
H	5.540500000	0.163400000	-11.665000000
H	5.520800000	-1.527400000	-11.147700000
H	4.098200000	-0.500400000	-10.886200000

Ag^{+}- DO4SMe

Ag	6.040600000	-0.776500000	-6.211100000
S	7.753500000	-2.940700000	-6.180800000
S	3.771000000	-4.266800000	-10.476600000
S	4.652500000	-1.867100000	-4.102100000
S	8.450400000	1.960700000	-1.526200000
N	7.815900000	-0.125500000	-7.947900000
N	4.857000000	-0.750200000	-8.517800000
N	4.051900000	0.821100000	-5.951300000
N	7.009000000	1.486800000	-5.410100000
C	7.200600000	-0.295800000	-9.265300000
H	7.319600000	-1.340300000	-9.569000000
H	7.739300000	0.287600000	-10.031500000

C	5.717800000	0.077400000	-9.377100000
H	5.612300000	1.094000000	-8.982000000
C	3.566000000	-0.136500000	-8.226600000
H	2.945600000	-0.906400000	-7.751400000
H	3.026200000	0.149000000	-9.145000000
C	3.573500000	1.102600000	-7.323100000
H	4.294600000	1.816300000	-7.736500000
C	4.568800000	2.034000000	-5.315200000
H	4.564400000	1.882900000	-4.231500000
H	3.893200000	2.889400000	-5.487400000
C	5.973900000	2.479600000	-5.737400000
H	5.977300000	2.535800000	-6.831800000
C	8.226600000	1.636400000	-6.199800000
H	8.984400000	0.986800000	-5.744400000
H	8.639100000	2.657300000	-6.136800000
C	8.132800000	1.297100000	-7.692000000
H	7.289900000	1.857500000	-8.111500000
C	8.994000000	-0.978500000	-7.814000000
H	9.575000000	-0.633100000	-6.953100000
H	9.661300000	-0.896000000	-8.693200000
C	8.708700000	-2.463100000	-7.629400000
H	8.156700000	-2.883300000	-8.479300000
H	9.667200000	-3.002200000	-7.605700000
C	8.996700000	-2.858000000	-4.885600000
H	9.787800000	-3.594400000	-5.073100000
H	8.490400000	-3.127900000	-3.951700000
H	9.429600000	-1.860500000	-4.774700000
C	4.705500000	-2.157100000	-8.922100000
H	4.470000000	-2.726000000	-8.014700000
H	5.676900000	-2.527200000	-9.261000000
C	3.667900000	-2.532200000	-9.987400000
H	2.640600000	-2.320600000	-9.661700000
H	3.823700000	-1.985200000	-10.922100000
C	2.961300000	-5.091100000	-9.100700000
H	3.535600000	-5.037900000	-8.170000000
H	2.883600000	-6.146500000	-9.388800000
H	1.944800000	-4.707200000	-8.949900000
C	3.012700000	0.210300000	-5.126700000
H	2.469000000	-0.517000000	-5.738000000
H	2.266100000	0.955000000	-4.790400000
C	3.506600000	-0.498800000	-3.872300000
H	4.022700000	0.187500000	-3.189500000
H	2.633900000	-0.867700000	-3.313600000
C	3.538300000	-3.190700000	-4.588400000
H	4.162800000	-4.074200000	-4.763400000
H	2.984600000	-2.965200000	-5.503500000
H	2.842300000	-3.417800000	-3.771600000
C	7.316500000	1.317500000	-3.980700000
H	7.714700000	0.303100000	-3.859800000
H	6.379100000	1.336400000	-3.417900000
C	8.291600000	2.282700000	-3.295600000
H	9.292500000	2.255100000	-3.746300000
H	7.947700000	3.320200000	-3.348400000
C	9.565000000	0.552200000	-1.504600000
H	9.789200000	0.364600000	-0.447400000
H	10.508700000	0.784100000	-2.013600000
H	9.116600000	-0.356500000	-1.919300000
C	2.202900000	1.776200000	-7.411800000
H	2.047100000	2.167300000	-8.424800000
H	1.382300000	1.080600000	-7.200100000
H	2.114400000	2.624000000	-6.724200000
C	6.190000000	3.909500000	-5.237800000
H	5.986200000	4.006500000	-4.165400000
H	7.206600000	4.263900000	-5.434500000
H	5.505700000	4.590900000	-5.760400000
C	9.387900000	1.823900000	-8.390100000
H	10.310000000	1.466400000	-7.916600000
H	9.419900000	1.543700000	-9.448300000
H	9.402300000	2.920000000	-8.348200000
C	5.357700000	0.159200000	-10.861900000
H	5.904800000	0.988400000	-11.329400000
H	5.630200000	-0.753900000	-11.402900000
H	4.291600000	0.353300000	-11.015700000

Table S2. ^{1}H-NMR signals of Ag^{+}-DO4S. Chemical shift values (ppm) are given and the following information is reported in brackets: multiplicity, area (if applicable) and proton assignation.

Species	pD	^{1}H-NMR signals
**AgL^{+}**	10.3<br>8.9<br>7.2<br>6.0	2.22 (s, 12, SCH_3); 2.75 (s broad, 24, NCH_2); 2.84 (t, 8, SCH_2)
**AgL^{+} (58 %) + $AgHL^{2+}$ (41%)**	4.4	AgL^{+}: 2.20 (s, 12, SCH_3); 2.73 (s broad, 24, NCH_2); 2.82 (t, 8, SCH_2);<br>$AgHL^{2+}$: 2.30 (s, 12, SCH_3); 2.97 (s broad, 24, NCH_2); 3.04 (t, 8, SCH_2)
**AgL^{+} (37 %) + $AgHL^{2+}$ (62%)**	3.6	AgL^{+}: 2.20 (s, 12, SCH_3); 2.72 (s broad, 24, NCH_2); 2.82 (s broad, 8, SCH_2)<br>$AgHL^{2+}$: 2.30 (s, 12, SCH_3); 2.97 (s broad, 24, NCH_2); 3.04 (s broad, 8, SCH_2)
**H_2L^{2+} (14 %) + $AgHL^{2+}$ (86%)**	2.1	H_2L^{2+}: 2.20 (s, SCH_3)<br>$AgHL^{2+}$: 2.32 (s, 12, SCH_3); 2.98 (s broad, 8, SCH_2); 3.05 (s broad, 24, NCH_2)

Table S3. ^{1}H-NMR signals of Ag^{+}-DO3S. Chemical shift values (ppm) are given and the following information is reported in brackets: multiplicity, area (if applicable) and proton assignation.

Species	pD	^{1}H-NMR signals
**AgL^{+}**	10.1<br>9.1<br>7.8	2.19 (s, 3, SCH_3); 2.23 (s, 6, SCH_3); 2.44-2.90 (m, broad, 28, SCH_2 + NCH_2)
**AgL^{+} + $AgHL^{2+}$**	6.6	2.30 (s, 12, SCH_3); 2.46-3.05 (m broad, 28, SCH_2 + NCH_2)
**$AgHL^{2+}$**	5.4<br>4.2	2.33 (s, 9, SCH_3); 2.46-3.05 (m, broad, 28, SCH_2 + NCH_2)
**$AgHL^{2+}$(92%) + H_2L^{2+} (9%)**	3.3	$AgHL^{2+}$: 2.33 (s, 9, SCH_3); 2.46-3.05 (m, broad, 28, SCH_2 + NCH_2)<br>H_2L^{2+}: 2.17 (s, SCH_3); 2.21 (s, SCH_3)

Table S4. ^{1}H-NMR signals of Ag^{+}-DO3SAm. Chemical shift values (ppm) are given and the following information is reported in brackets: multiplicity, area and proton assignation.

Species	pD	^{1}H-NMR signals
**AgL^{+}**	10.3<br>8.1<br>3.7	2.39 (s, 6, SCH_3); 2.45 (s, 3, SCH_3); 2.58-3.28 (m, broad, 31, SCH_2 + NCH_2 + NCH_3); 2.73 (s, 2, $\underline{CH_2}CONHCH_3$)
**$AgHL^{2+}$**	2.4<br>2.0	2.35 (s, 9, SCH_3); 2.66-3.25 (m, broad, 31, SCH_2 + NCH_2 + NCH_3); 3.53 (s, 2, $\underline{CH_2}CONHCH_3$)

Table S5. ^{1}H-NMR signals of Ag^{+}-DO2A2S. Chemical shift values (ppm) are given and the following information is reported in brackets: multiplicity, area and proton assignation.

Species	pD	^{1}H-NMR signals
**AgL^{-}**	11.2<br>8.6<br>7.8	2.30 (s, 6, SCH_3); 2.60 + 2.72 + 2.80 + 2.95 (s, broad, 24, NCH_2); 2.90 (t, 4, SCH_2); 3.38 (s, 4, $\underline{CH_2}COOH$)
**AgHL**	4.6	2.30 (s, 6, SCH_3); 2.80 + 3.10 (s, broad, 24, NCH_2); 2.92 (t, 4, SCH_2); 3.55 (s, broad, 4, $\underline{CH_2}COOH$)
**AgH_2L^{+}**	3.5	2.30 (s, 6, SCH_3); 2.80 + 3.15 (s, broad, 24, NCH_2); 2.92 (t, 4, SCH_2); 3.65 (s, broad, 4, $\underline{CH_2}COOH$)

Table S6. ^{1}H-NMR signals of Ag^{+}-DO4S4Me. Chemical shift values (ppm) are given and the following information is reported in brackets: multiplicity, area (if applicable) and proton assignation.

Species	pH	^{1}H-NMR signals
**AgL^{+}**	≥ 7.55<br>3.74<br>2.86	0.93 (d, 12, CH_3); 2.39 (s, 3, SCH_3); 2.40 (s, 9, SCH_3); 2.68 (m, 2, NCH_2 ring), 2.82 (m, 8, NCH_2 ring + SCH_2), 2.90 (m, 2, NCH_2 side arm), 3.01 (m, 6, NCH_2 side arm), 3.16 (m, 6, SCH_2), 3.23 (3, NCH ring), 3.31 (m, 1, NCH ring)
**AgL^{+} + $AgHL^{2+}$**	2	AgL^{+}: 0.91 (d, 12, CH_3); 2.40 (s, 3, SCH_3); 2.44 (s, 9, SCH_3); 2.67 (m, 2, NCH_2 ring), 2.80 (m, 8, NCH_2 ring + SCH_2), 2.88 (m, 2, NCH_2 side arm), 3.09 (m, 6, NCH_2 side arm), 3.16 (m, 9, SCH_2 + NCH ring), 3.29 (m, 1, NCH ring)<br>$AgHL^{2+}$: 1.08 (d, CH_3), 2.37 (s, SCH_3), 3.52 (s, CH)

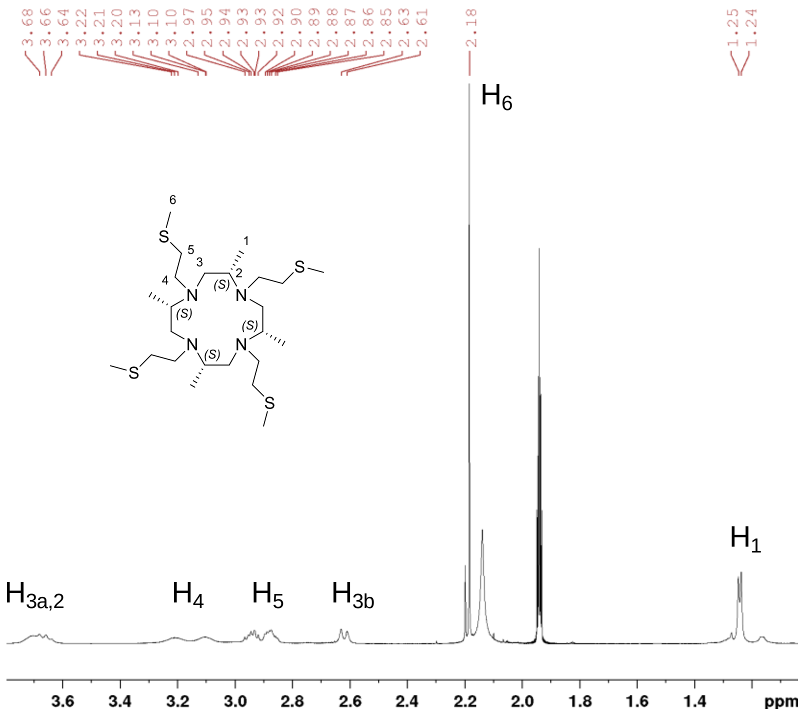

Figure S1: ^{1}H NMR spectrum of DO4S4Me (600 MHz, 25°C, MeCN). Signal assignation according to COSY and HMQC spectra (Figures S2 and S3).

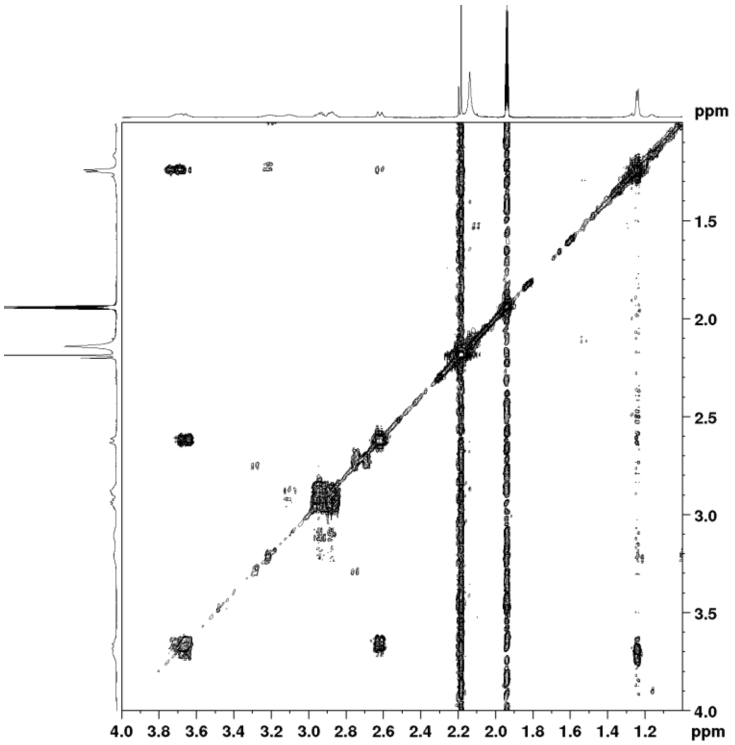

Figure S2: COSY spectrum of DO4S4Me (600 MHz, 25°C, MeCN).

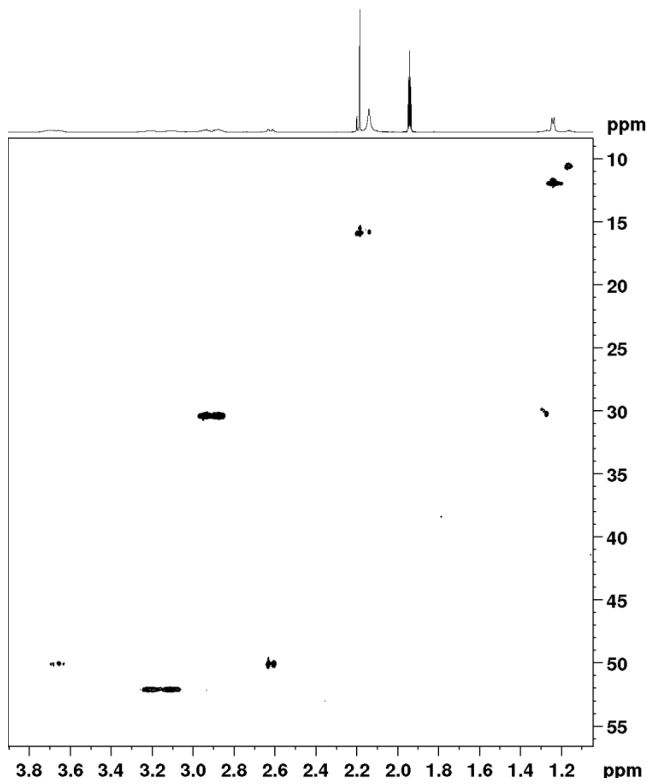

Figure S3: HMQC spectrum of DO4S4Me (600 MHz, 25°C, MeCN).

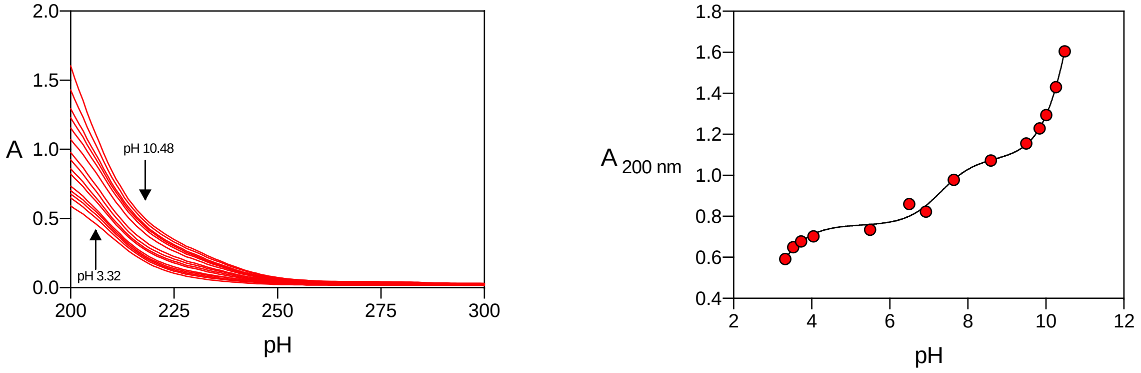

Figure S4. A) UV-Vis spectra obtained in a spectrophotometric titration of DO4S4Me ($C_{DO4S4Me}$ = 1.14·10^{-4} mol/L); B) experimental points and fitting line of absorbance *vs.* pH at λ = 200 nm. Acidity constants results: pK_{H2L} = 7.30 ± 0.50 and pK_{HL} = 10.85 ± 0.88.

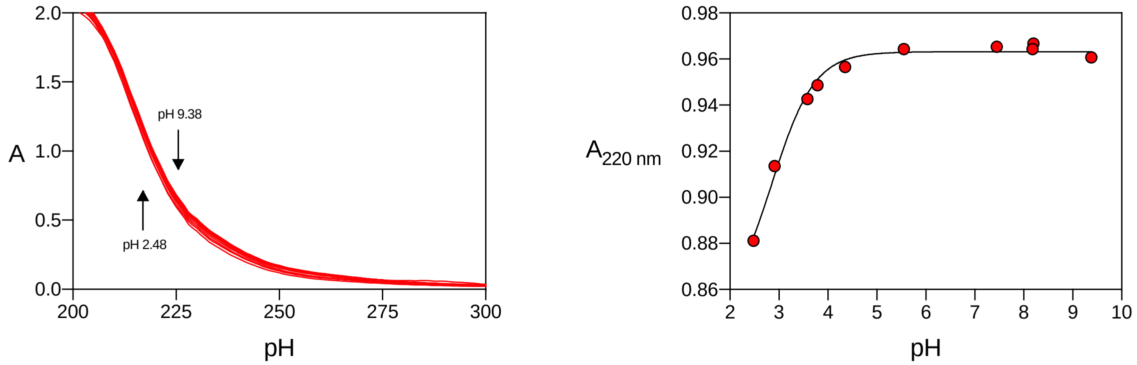

Figure S5. A) UV-Vis spectra of solutions containing Ag^{+} and DO4S4Me (C_{Ag} = $C_{DO4S4Me}$ = 1.14·10^{-4} mol/L, 2.48 ≤ pH ≤ 9.38); B) experimental points and fitting line of absorbance *vs.* pH at λ = 220 nm.

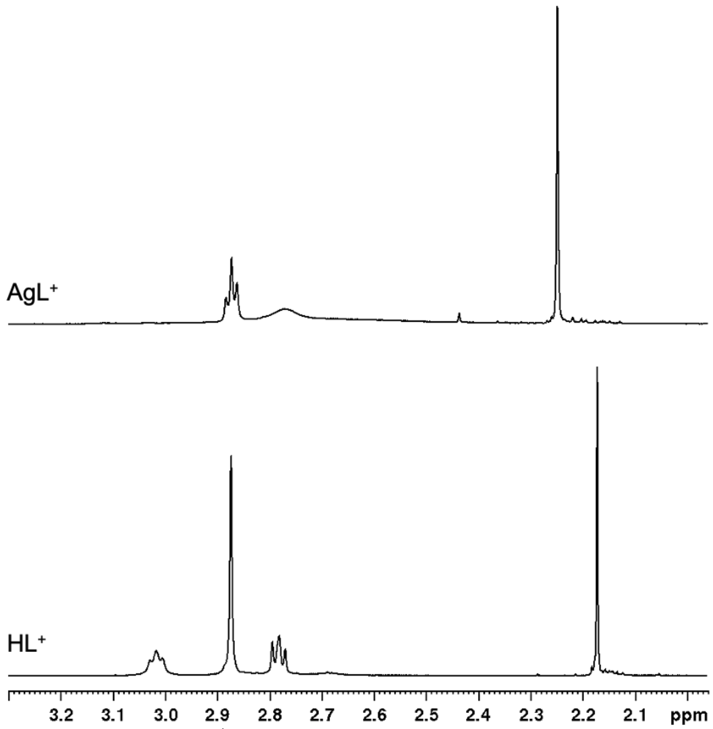

Figure S6. Comparison between the ^{1}H NMR spectra (600 MHz, 25°C, D_2O) of AgL^+ and free ligand at pD ~ 10 (L = DO4S; data for the complex and for free ligand were taken respectively from Figure 5 and from [1] - see reference list at the end of this file).

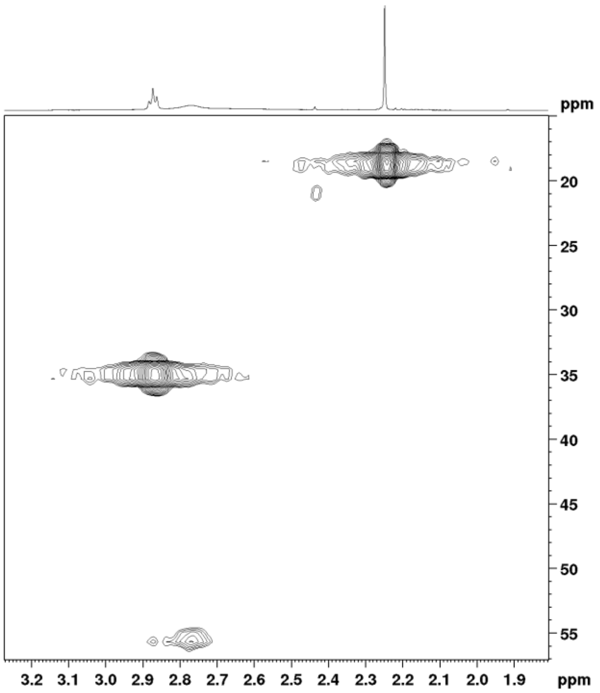

Figure S7. HMQC spectrum of the AgL^+ complex formed by DO4S (pD 10.3; see also caption of Figure 5).

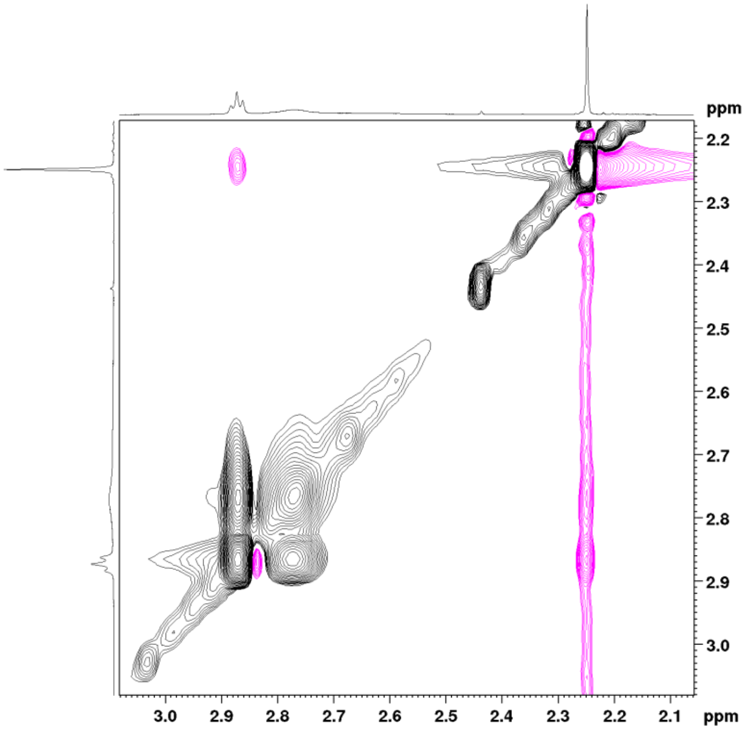

Figure S8. NOESY spectrum of the AgL^+ complex formed by DO4S (pD 10.3; see also caption of Figure 5).

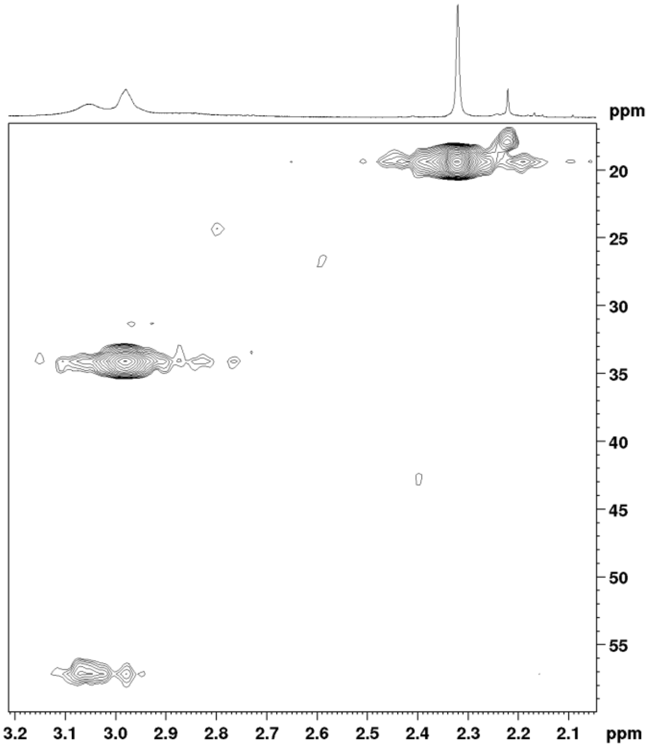

Figure S9. HMQC spectrum of the $AgLH^{2+}$ complex formed by DO4S (pD 2.1; see also caption of Figure 5).

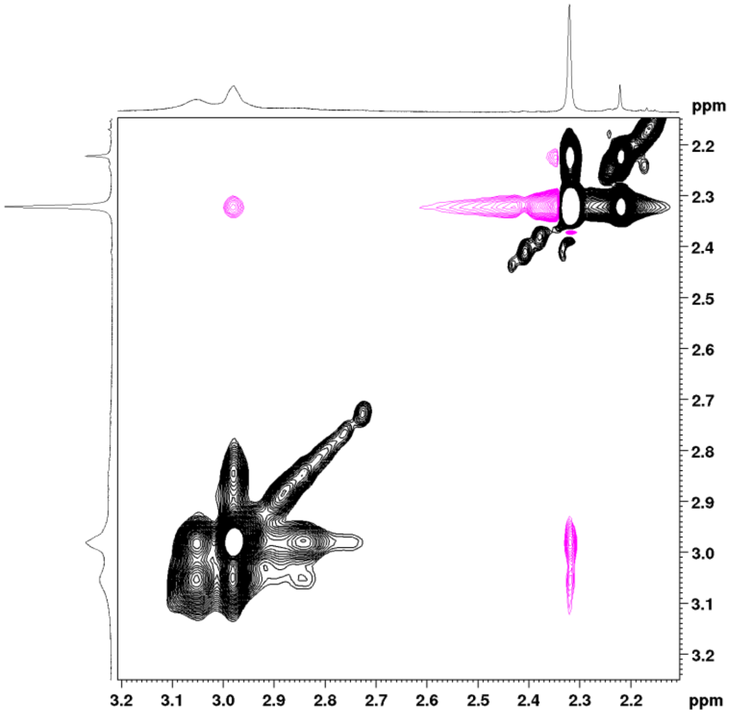

Figure S10. NOESY spectrum of the $AgLH^{2+}$ complex formed by DO4S (pD 2.1; see also caption of Figure 5).

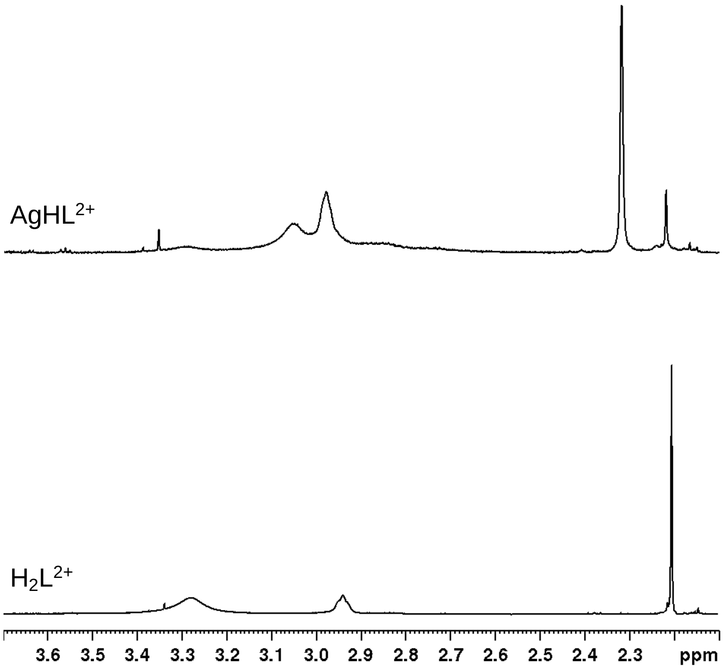

Figure S11. Comparison between the ^1H NMR spectra (600 MHz, 25°C, D_2O) of $AgHL^{2+}$ and free ligand at pD ~ 2 (L = DO4S; data for the complex and for free ligand were taken respectively from Figure 5 and from [1] - see reference list at the end of this file).

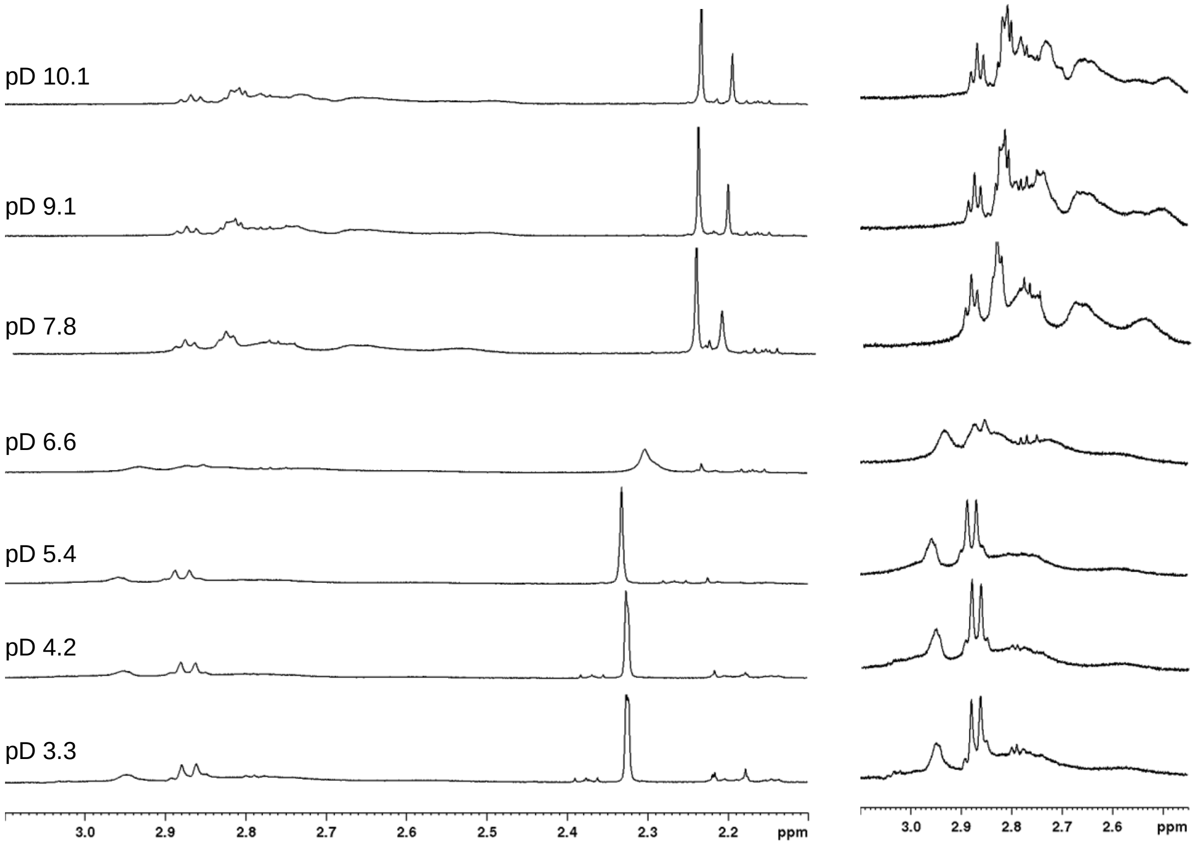

Figure S12. ^{1}H NMR spectra solutions containing Ag^{+} and DO3S (600 MHz, 25°C, D_2O, $C_{Ag} = 9.3 \cdot 10^{-4}$ M, $C_{DO3S} = 9.4 \cdot 10^{-4}$ M,) at various pD values. The spectral region in the range of 2.50-3.10 ppm was enlarged for clarity.

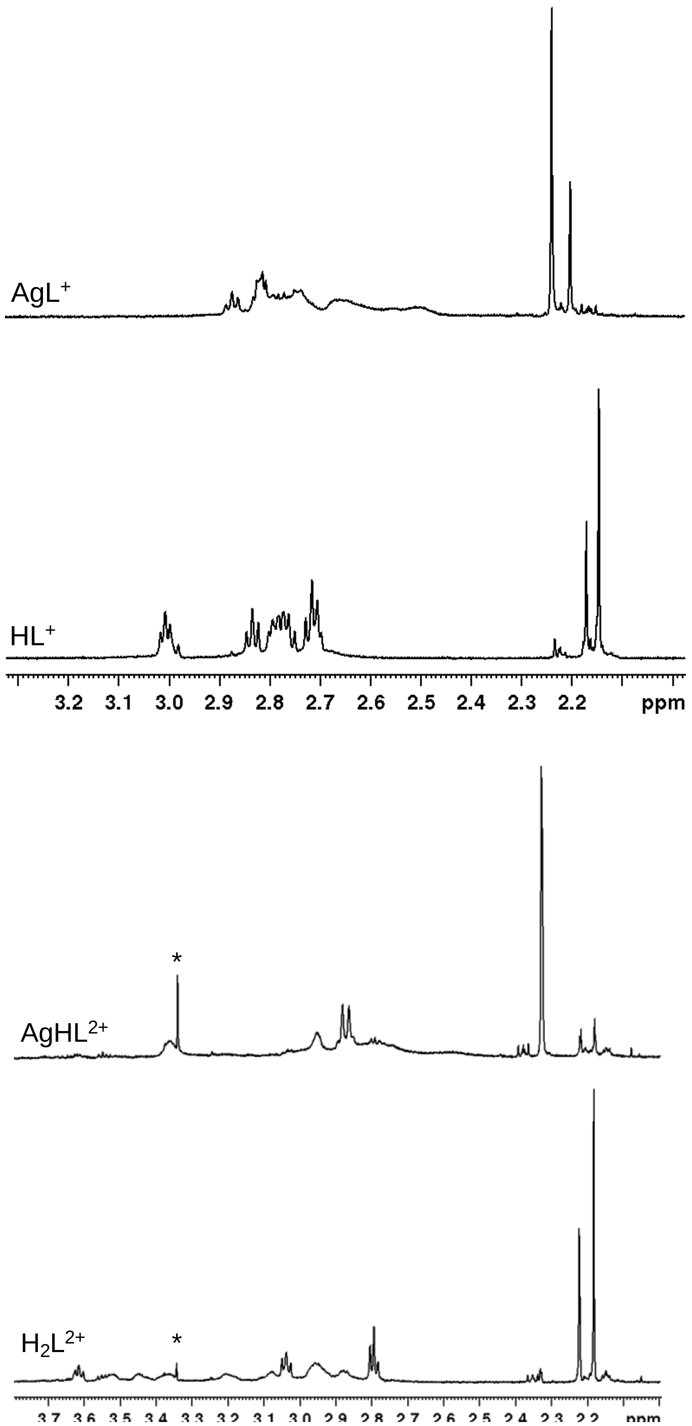

Figure S13. Comparison between the ^{1}H NMR spectra (600 MHz, 25°C, D_2O) of AgL^+ and free ligand at pD ~ 9 (above) and $AgHL^{2+}$ and free ligand at pD ~ 3 (below; L = DO3S; data for the complex and for free ligand were taken respectively from Figure S12 and from [1] - see reference list at the end of this file). The signals marked with an asterisk are related to MeOH impurities.

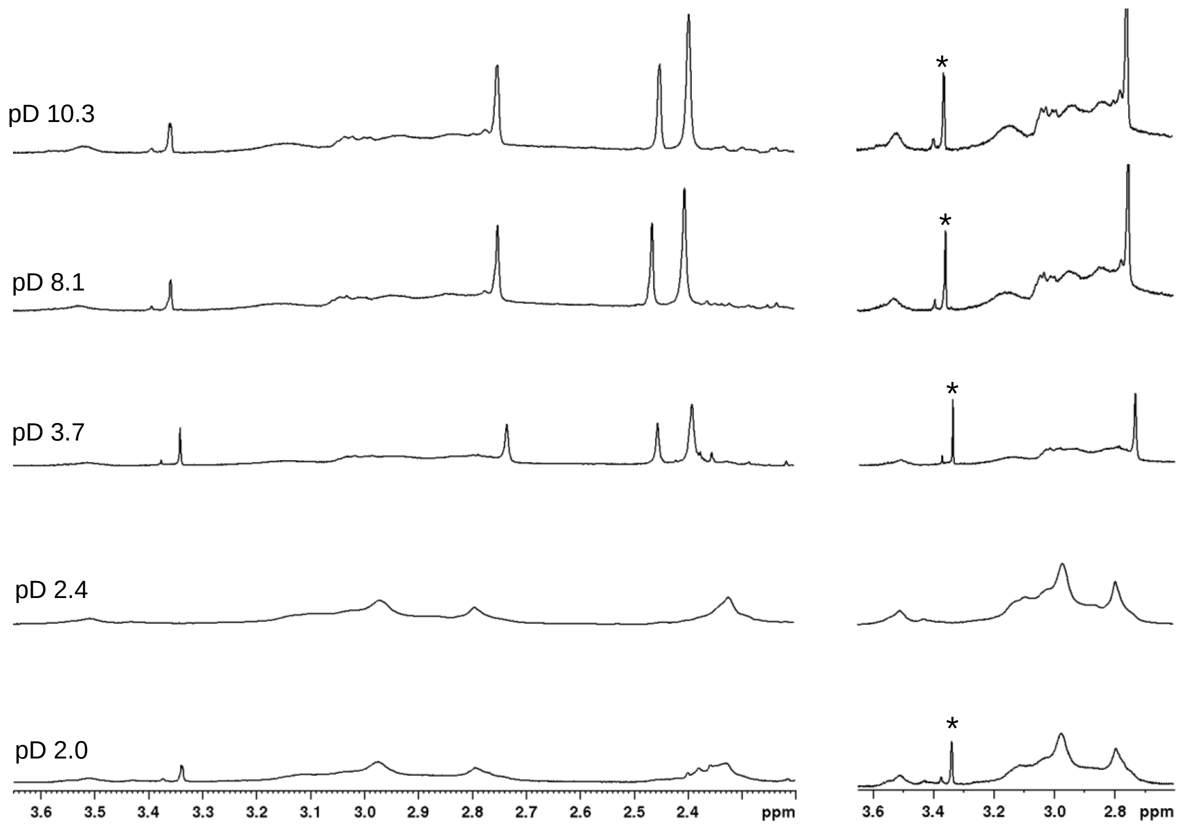

Figure S14. ^{1}H NMR spectra of solutions containing Ag^{+} and DO3SAm (400 MHz, 25°C, D_2O, C_{Ag} = 8.6·10^{-4} M C_{DO3SAm} = 8.5·10^{-4} M) at various pD values. The spectral region in the range of 2.70-3.60 ppm has been enlarged for clarity. The signals marked with an asterisk are related to MeOH impurities.

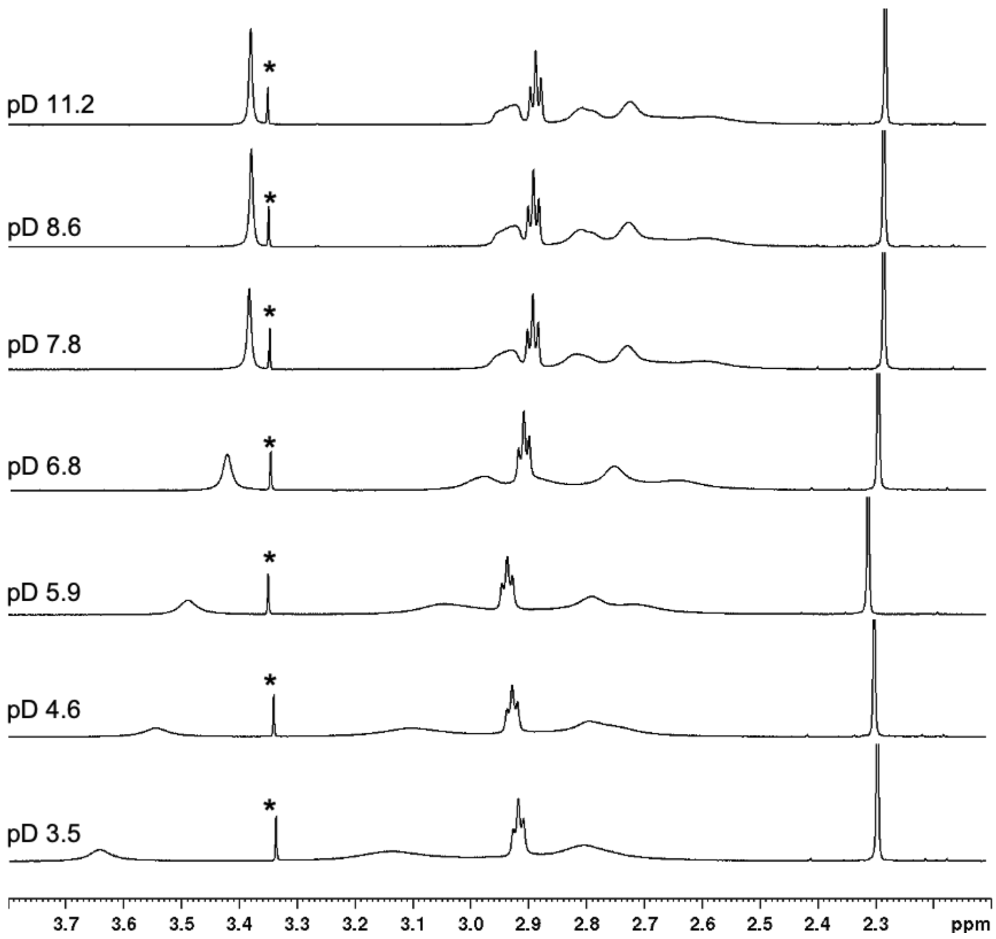

Figure S15. ^{1}H NMR spectra of solutions containing Ag^+ and DO2A2S (600 MHz, D_2O, C_{Ag} = C_{DO2A2S} = $2.0 \cdot 10^{-3}$ M,) at various pD values. The signals marked with an asterisk are related to MeOH impurities.

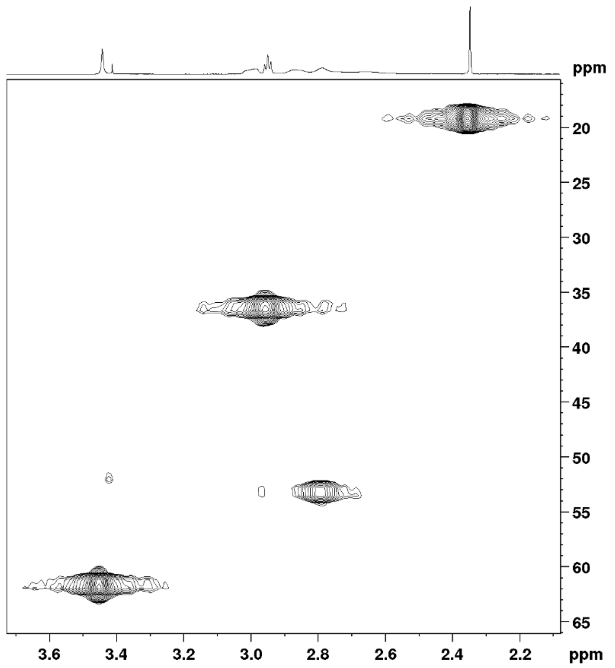

Figure S16. HMQC spectrum of the AgL^{-} complex formed by DO2A2S (pD 11.2; see also caption of Figure S15).

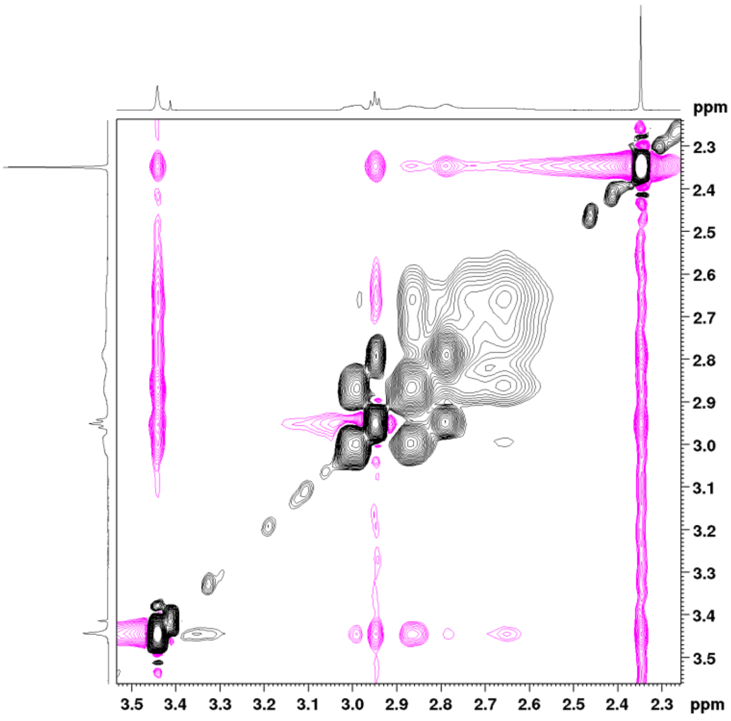

Figure S17. NOESY spectrum of the AgL^{-} complex formed by DO2A2S (pD 11.2; see also caption of Figure S15).

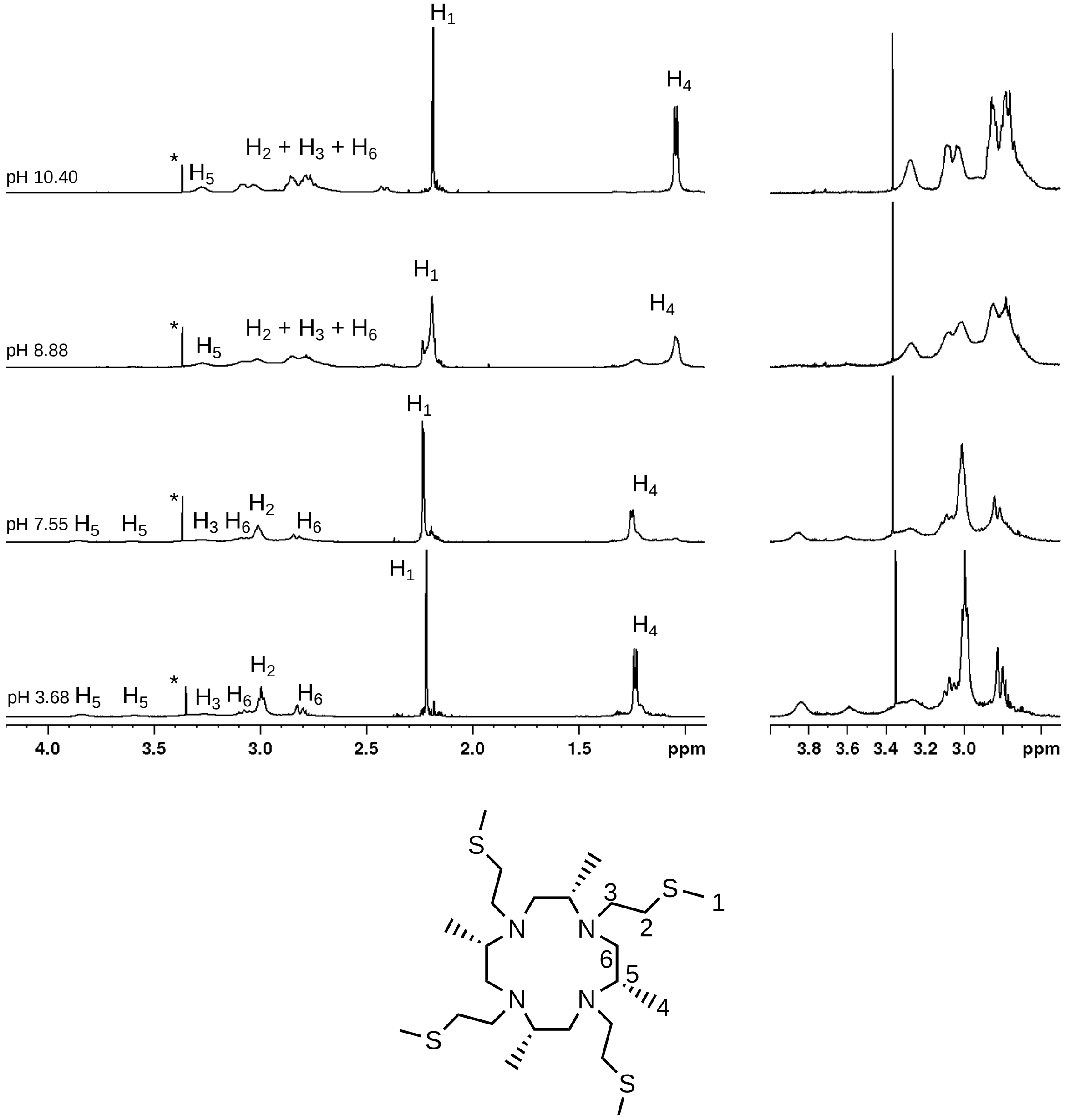

Figure S18. ^{1}H NMR spectra solutions containing DO4S4Me (600 MHz, 25°C, H_2O + 10% D_2O, $C_{DO4S4Me}$ = $1.0{\cdot}10^{-3}$ M) at various pD values and signal assignation, according to integration values and COSY spectra (Figures S19 and S20). The signals marked with an asterisk are related to MeOH impurities.

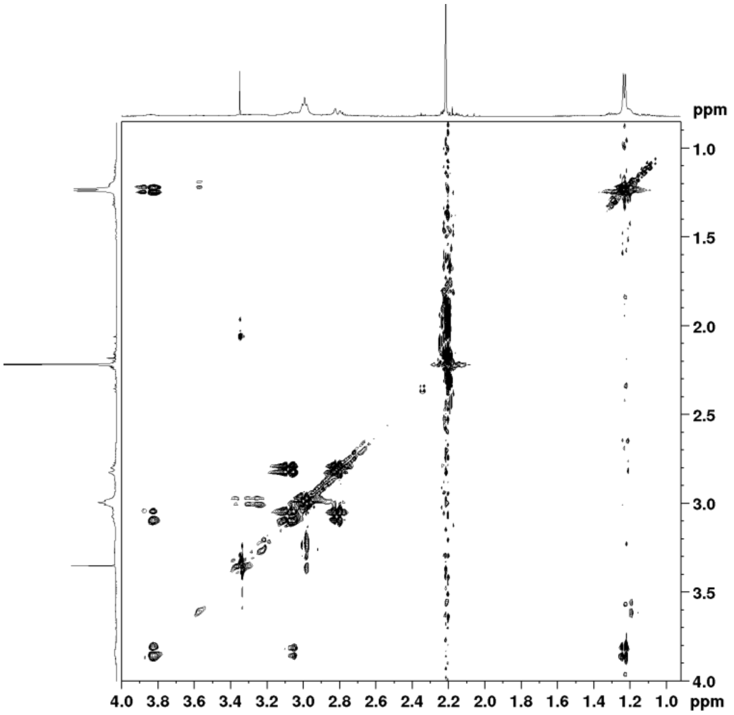

Figure S19: COSY spectrum of diprotonated DO4S4Me (pH 3.68; see caption of Figure S18).

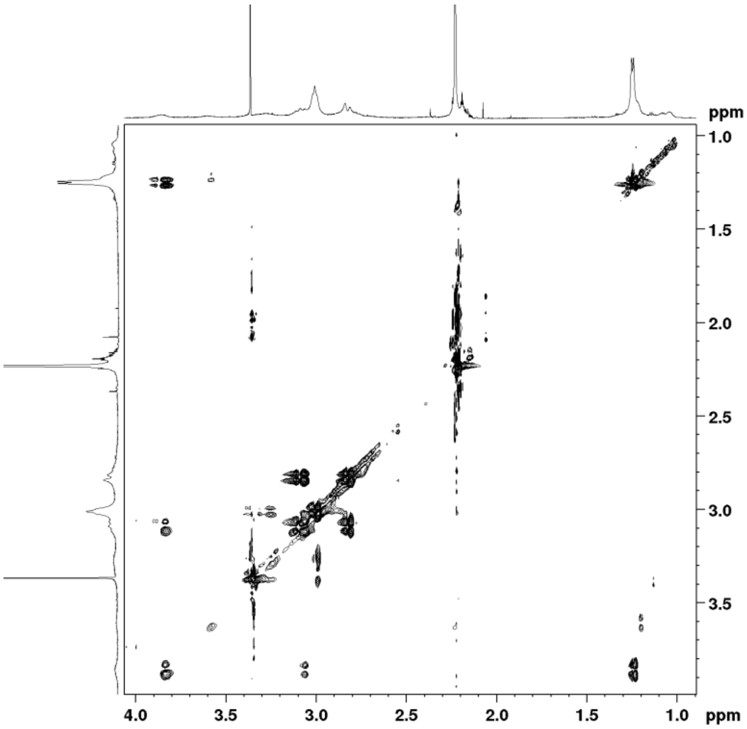

Figure S20: COSY spectrum of monoprotonated DO4S4Me (pH 7.55; see caption of Figure S18).

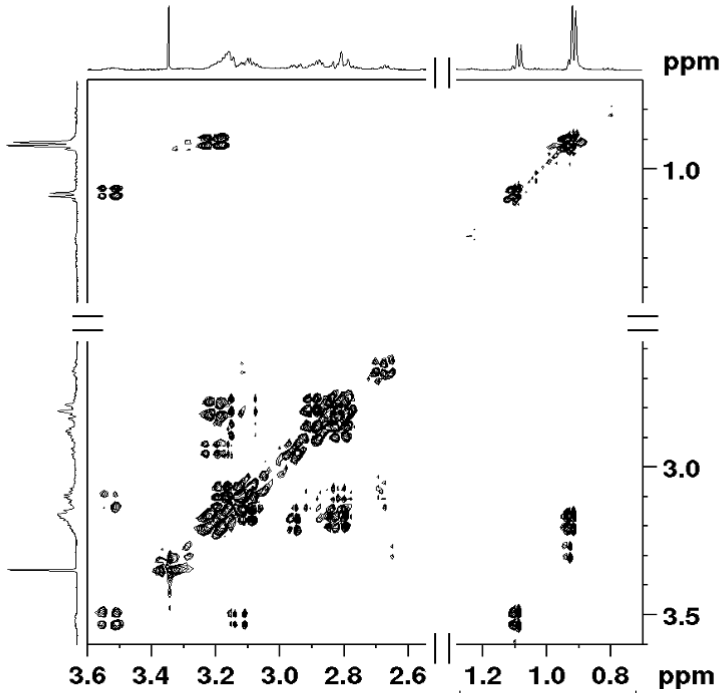

Figure S21. COSY spectrum of the mixture of AgL^+ and $AgHL^+$ complexes formed by DO4S4Me (pH 2; see also caption of Figure 6). Only the 0.70-1.30 and 2.60-3.60 ppm regions have been enlarged for clarity.

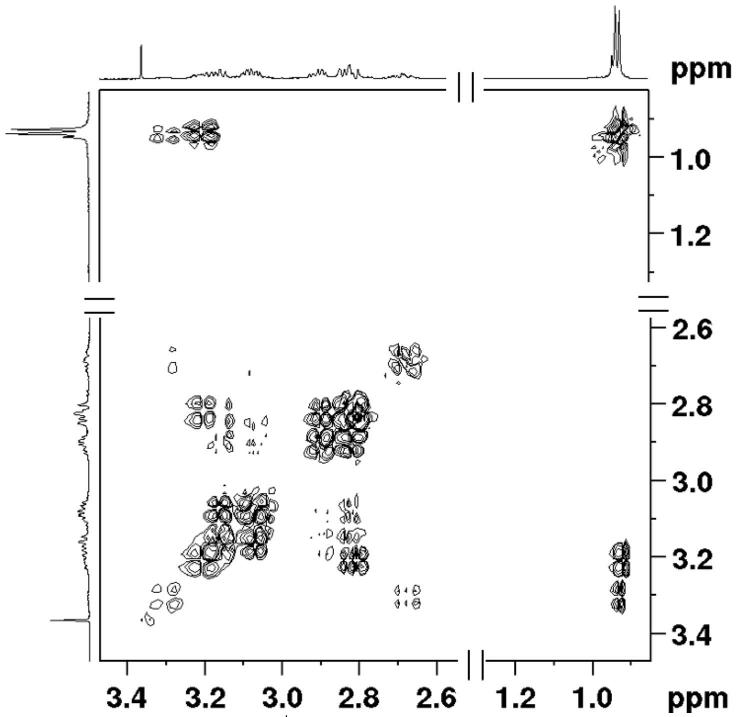

Figure S22. COSY spectrum of the AgL^+ complex formed by DO4S4Me (pH 7.05; see also caption of Figure 6). Only the 0.70-1.30 and 2.60-3.60 ppm regions have been enlarged for clarity.

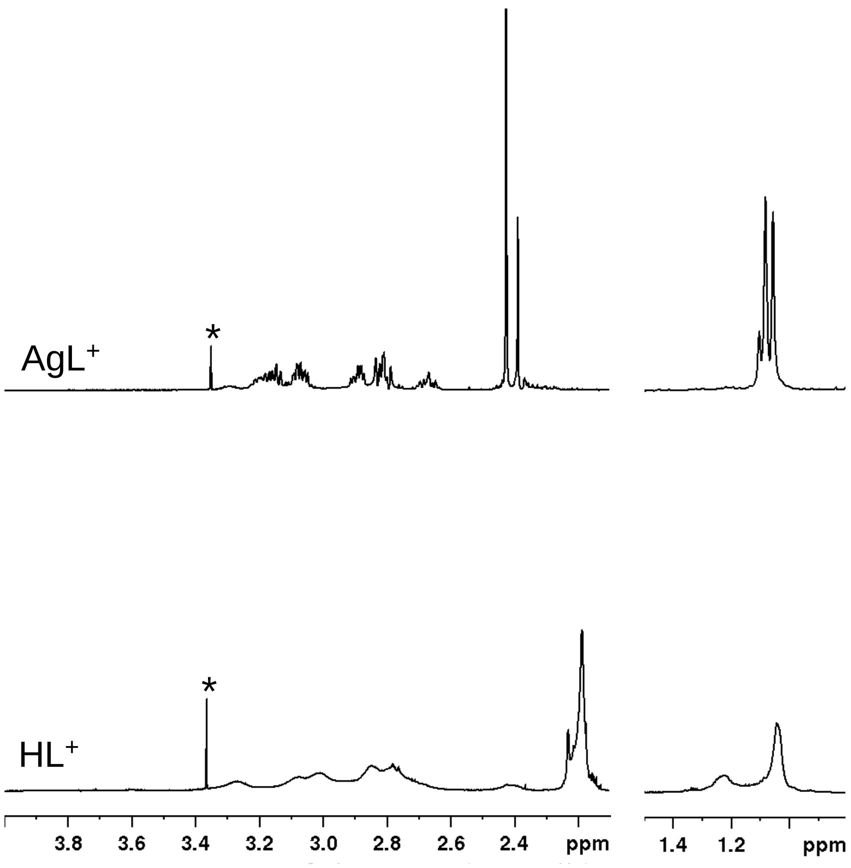

Figure S23. Comparison between the ^{1}H NMR spectra (600 MHz, 25°C, H_2O + 10% D_2O) of AgL^+ and free ligand at pH ~ 9 (L = DO4S4Me). The signals marked with an asterisk are related to MeOH impurities.

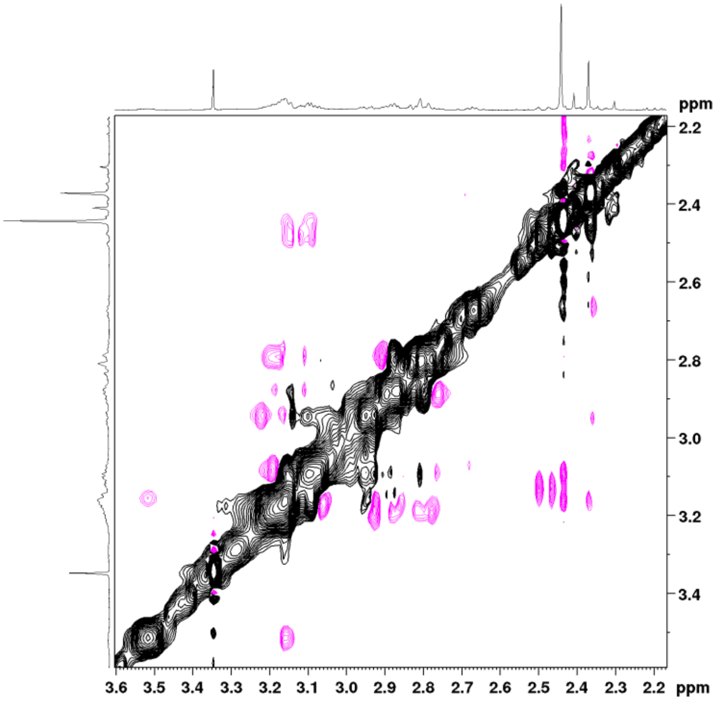

Figure S24. NOESY spectrum of mixture of $AgHL^{2+}$ and AgL^{+} complex formed by DO4S4Me (pH 2; see also caption of Figure 6). Only the 2.20-3.60 ppm region has been enlarged for clarity.

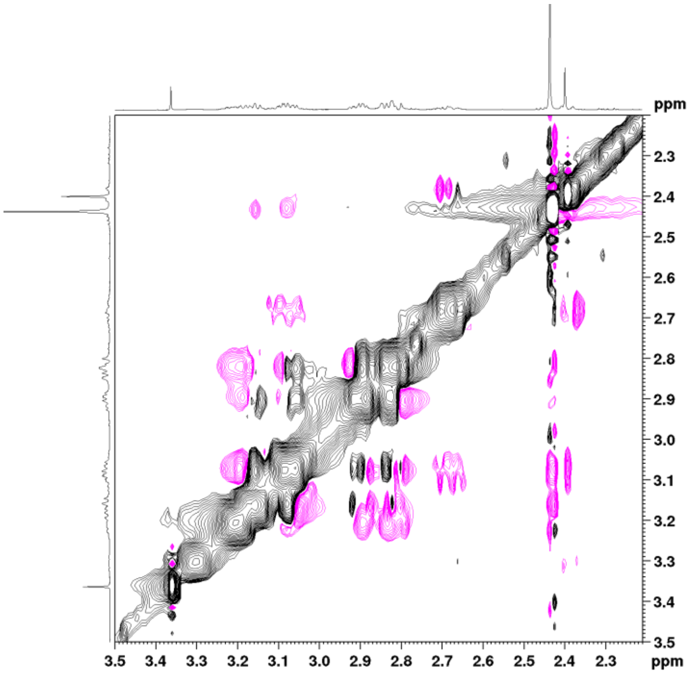

Figure S25. NOESY spectrum of the AgL^{+} complex formed by DO4S4Me (pH 7.05; see also caption of Figure 6). Only the 2.20-3.50 ppm region has been enlarged for clarity.

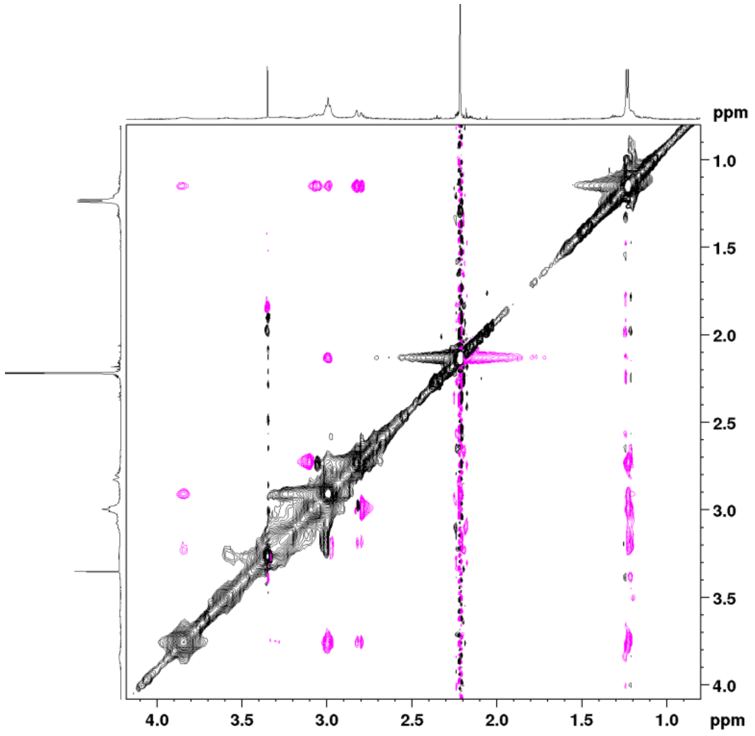

Figure S26: NOESY spectrum of diprotonated DO4S4Me (pH 3.68).

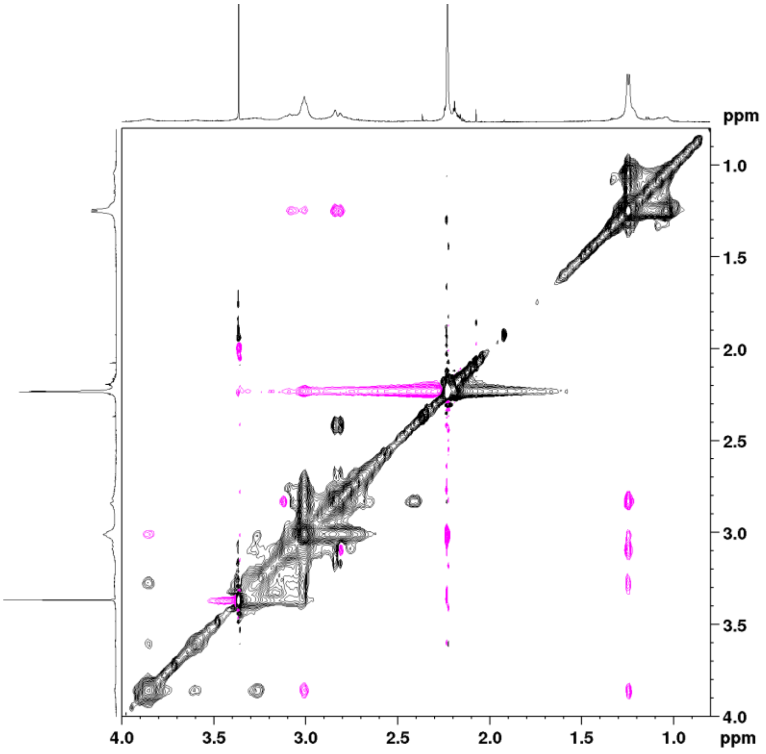

Figure S27: NOESY spectrum of monoprotonated DO4S4Me (pH 7.55).

Supplementary reference

(1) Tosato, M.; Verona, M.; Doro, R.; Dalla Tiezza, M.; Orian, L.; Andrighetto, A.; Pastore, P.; Marzaro, G.; Di Marco, V. Toward Novel Sulphur-Containing Derivatives of Tetraazacyclododecane: Synthesis, Acid-Base Properties, Spectroscopic Characterization, DFT Calculations, and Cadmium(II) Complex Formation in Aqueous Solution. *New J. Chem.* **2020**, *44* (20), 8337–8350.
